# MicroDNA levels are dependent on MMEJ, repressed by c-NHEJ pathway, and stimulated by DNA damage

**DOI:** 10.1093/nar/gkab984

**Published:** 2021-10-30

**Authors:** Teressa Paulsen, Pumoli Malapati, Yoshiyuki Shibata, Briana Wilson, Rebeka Eki, Mouadh Benamar, Tarek Abbas, Anindya Dutta

**Affiliations:** Department of Biochemistry and Molecular Genetics, University of Virginia School of Medicine, Charlottesville, VA 22908, USA; Department of Oncology, University of Oxford, Oxford OX3 7DQ, UK; Department of Biochemistry and Molecular Genetics, University of Virginia School of Medicine, Charlottesville, VA 22908, USA; Department of Biochemistry and Molecular Genetics, University of Virginia School of Medicine, Charlottesville, VA 22908, USA; Department of Genetics, University of Alabama at Birmingham, Birmingham, AL 35294-0024, USA; Department of Biochemistry and Molecular Genetics, University of Virginia School of Medicine, Charlottesville, VA 22908, USA; Department of Biochemistry and Molecular Genetics, University of Virginia School of Medicine, Charlottesville, VA 22908, USA; Department of Radiation Oncology, University of Virginia School of Medicine, Charlottesville, VA 22908, USA; Department of Biochemistry and Molecular Genetics, University of Virginia School of Medicine, Charlottesville, VA 22908, USA; Department of Radiation Oncology, University of Virginia School of Medicine, Charlottesville, VA 22908, USA; Department of Biochemistry and Molecular Genetics, University of Virginia School of Medicine, Charlottesville, VA 22908, USA; Department of Radiation Oncology, University of Virginia School of Medicine, Charlottesville, VA 22908, USA; Department of Biochemistry and Molecular Genetics, University of Virginia School of Medicine, Charlottesville, VA 22908, USA; Department of Genetics, University of Alabama at Birmingham, Birmingham, AL 35294-0024, USA

## Abstract

Extrachromosomal circular DNA (eccDNA) are present within all eukaryotic organisms and actively contribute to gene expression changes. MicroDNA (200-1000bp) are the most abundant type of eccDNA and can amplify tRNA, microRNA, and novel si-like RNA sequences. Due to the heterogeneity of microDNA and the limited technology to directly quantify circular DNA molecules, the specific DNA repair pathways that contribute to microDNA formation have not been fully elucidated. Using a sensitive and quantitative assay that quantifies eight known abundant microDNA, we report that microDNA levels are dependent on resection after double-strand DNA break (DSB) and repair by Microhomology Mediated End Joining (MMEJ). Further, repair of DSB without resection by canonical Non-Homologous End Joining (c-NHEJ) diminishes microDNA formation. MicroDNA levels are induced locally even by a single site-directed DSB, suggesting that excision of genomic DNA by two closely spaced DSB is not necessary for microDNA formation. Consistent with all this, microDNA levels accumulate as cells undergo replication in S-phase, when DNA breaks and repair are elevated, and microDNA levels are decreased if DNA synthesis is prevented. Thus, formation of microDNA occurs during the repair of endogenous or induced DNA breaks by resection-based DNA repair pathways.

## INTRODUCTION

The field of extrachromosomal circular DNA (eccDNA) has recently burgeoned due to the increasing evidence of both the prevalence and active role of eccDNA in normal tissues and cancers (1–8). The eccDNA in different cancer cell lines are very diverse and hence have been categorized into groups based on size and characteristics (3). The larger eccDNA (>10 kb), termed double minutes, have been well categorized and studied due to their contribution to cancer growth by sustaining and amplifying full gene sequences through circular molecules that contain promoters, enhancers and replication origin sequences. They are often composed of DNA segments from multiple loci (multilocular) and their massive size enables these molecules to be visible through fluorescent microscopy during normal karyotyping (9–11). Their formation has been tied to chromothripsis, as the long eccDNA contain a number of fragments from different parts of the genome. Though these double minutes have been the most characterized, they are the least abundant type of eccDNAs in both normal and cancer cells. The eccDNAs of smaller sizes (<10 kb) make up >99% of the eccDNA population in normal and cancer cells (5,12–15). These eccDNA molecules, termed microDNA, are highly heterogeneous, are usually derived from a single locus (monolocular) and have been found to express microRNA and si-like-RNA which are capable of affecting gene expression (5). They are occasionally long enough to carry an oncogene, e.g. *EGFR* (1). The formation of these monolocular circles has been hypothesized to rely on some DNA repair mechanisms due to the variable presence of microhomology near the break points that are ligated to form the microDNA (3,8). In this study, we focus on the microDNA, excluding the very large eccDNA, double minutes.

The analysis of circular DNA has required the development of specialized adaptions of traditional techniques because of two major reasons: (i) circular DNA molecules are not accessible to sequencing because sequencing adaptors are usually ligated to the ends of linear DNA, (ii) circular DNA arise from genomic DNA and thus the methods must identify a unique junction sequence in order to distinguish an extrachromosomal circle from chromosomal DNA. To overcome these difficulties, tagmentation and rolling circle amplification (RCA) procedures have been utilized to create linear DNA molecules for DNA sequencing (2,4,6,15). However, these procedures distort the abundance of different eccDNA molecules because of relatively uncontrolled amplification (RCA) or because of experiment-specific variations in the linearization process (tagmentation or annealing of random hexamers to a circle) and do not allow a comparison of abundance under different experimental conditions.

In this study, we have adapted qPCR with specific primers to directly quantify microDNA levels such that they can be compared from experiment to experiment. This qPCR method utilized outward facing primers that amplify across the junction sequences of known microDNA, creating amplicons of comparable size with comparable efficiency regardless of microDNA size. Normalization methods allowed us to compare the relative levels of a set of microDNA under different experimental conditions. Using this method we have screened specific DNA repair pathways for their contribution to microDNA abundance.

We show that cellular microDNA abundance is decreased significantly when resection-based repair pathways, especially MMEJ, are compromised. We also show that double-strand breaks (DSBs) in the genome promote microDNA formation. The microDNA levels are increased significantly when the canonical-NHEJ (c-NHEJ) pathway is compromised, most likely because the repair of DSB is shunted towards resection-mediated MMEJ repair.

## MATERIALS AND METHODS

### EccDNA quantification

The microDNA was isolated from the various knock-out cell lines and treated cells using a HiSpeed midi-prep DNA isolation kit (Qiagen: 12643). The linear DNA was then digested using ATP-dependent plasmid safe DNase (Lucigen Catalog: E3110K). The remaining circular DNA was purified using DNA Clean & Concentrator-5 Kit (Genessee Catalog: 11-303). Then QPCR was performed using SYBR master mix (Life Technology Catalog: A25778) with the outward facing primers listed in Supplemental Tables S1 and S3.

We took several precautions to ensure that the results we obtained are generalizable to a group of representative microDNAs and corrected for the known variability in microDNA abundance between experiments because of the stochastic nature of their generation. For each experiment we measured the abundance of microDNA from at least 8 hot-spots, obtained the ratio of each microDNA to the mitochondrial DNA in the same cells in the same experiment, and normalized the microDNA : mitochondrial DNA ratio to that in the control cells. We thus obtained the average normalized abundance of the eight microDNA for that experiment (represented as one dot in the bar graphs). The experiment was repeated three to five times and the mean and standard error of the measurements plotted relative to that in control samples. Thus each bar in the figures in the paper has 3–5 independent dots to indicate the abundance of the group of representative microDNA in the 3–5 replicates and to demonstrate that the main conclusions are based on reproducible patterns.

The eccDNA candidates were chosen based on abundance levels determined from sequencing of RCA libraries and published in NCBI GEO (GSE68644 and GSE36088). Primers were designed to have similar Tms between all eight microDNA candidates, amplified comparable sized fragments independent of the lengths of the circles (Supplementary Tables S1 and S3) and for one set were experimentally shown to have comparable efficiency of amplification (Supplementary Figure S1B, C)

The abundance of each microDNA (measured by outward-facing primer (OF)), relative to mitochondrial DNA and normalized to the control sample was obtained by the formula:

$$\begin{eqnarray*} && {\rm Relative}\,\, {{\rm microDNA}}\,\, {\rm level}\,\, {\rm of}\,\, e{\rm xptl.sample} \\ && \quad =\frac{ 2^{{\rm average}({\rm Ct}_{{\rm Mt}\_{\rm exptl}})-{\rm Ct}_{{\rm OFPrimer}\_{\rm exptl}}}}{ 2^{{\rm average}({\rm Ct}_{{\rm Mt}\_{\rm control}})-{\rm Ct}_{{\rm OFPrimer}\_c{\rm ontrol}}}} \end{eqnarray*}$$

The average of eight microDNA was taken as the abundance of the microDNA in that experiment. Three (CRISPR induced DSB experiments) or five independent biological replicates (DNA damage, inhibitors, knock-out cell line experiments) were performed as indicated in the figure legends.

### Quantitative PCR

For quantification of purified microDNA we used *Power* SYBR Green PCR Master Mix (Fisher: 4368577) according to the manufacturer's instructions. The qPCR program was Denaturation at 95°C for 10 min; 40 cycles of Denaturation at 95°C for 15 s and Anneal/extension at 60°C for 90 s. Primer and amplicon sequences are included in the supplemental materials.

### Cell culture

HeLa cells were cultured in McCoy's medium;, 293A, 293T and U2OS cells were cultured in Dulbecco's modified Eagle's medium (DMEM); both supplemented with 10% fetal bovine serum, 100 U/ml penicillin and 100 μg/ml streptomycin in an environment containing 5% CO_2_ at 37°C. 293T cells were cultured in McCoy's medium supplemented with 10% fetal bovine serum, 100 U/ml penicillin and 100 μg/ml streptomycin in an environment containing 5% CO_2_ at 37°C.

The U2OS and DT40 knock out cell lines were obtained from the Dr. Abbas’ lab and Dr Pommier's lab respectively (15–17). Each cell line was grown from a single cell and was verified through PCR or Western Blot. The loss of the targeted proteins by Western blots has been reported in the papers reporting the engineering of the mutations (those that have not been reported previously are shown in Supplemental Figure S5A).

### Drug treatments

Drugs were added to the cell cultures at the following concentrations: NCS (200 ng/ml), cisplatin (2 uM), paclitaxel (10 nM), B02 (5 uM), AZD2461 (10 uM), MMS (200 uM), Mx (250 ug/ml), D-103 (30 uM), NSC16168 (0.5 uM). Cells were harvested after 48 h of treatment. The efficacy of the drugs is shown in Supplementary Figure S3.

### FACS analysis

Cells were fixed in 70% ethanol, washed, and then stained with propidium iodide (Thermo Fisher Catalog: P1304MP) according to the manufacturer's recommendation before subjecting to FACS.

### Transfection

The p413 plasmid expresses Cas9 together with a gRNA lacking the targeting site. The experimental plasmid has the chr22 gRNA targeting site inserted into the multiple cloning site of the gRNA. The plasmids were transfected in 293A cells using PEI using the manufacturer's instructions. Puromycin was added 48 hours after transfection for selection of transfected cells and microDNA was isolated 2 days after transfection.

### Statistical analysis

Cell line experiments were performed at least three independent times. In each biological replicate the microDNA candidate in each sample is normalized against mitochondrial DNA within the same sample. The normalized microDNA level is then divided by the normalized microDNA level in the control sample to get the fold-change. The fold changes of all eight microDNA in a given biological replicate are then averaged. Results show the mean of the biological replicates with the standard error of the replicates represented by error bars. For the comparison of two groups with a control group that has zero variance, a one sample *t*-test with a mu of 1 was performed to calculate the *P*-value. For comparisons of two groups with equal variance (as tested by an *F* test for equality of variance), a two sample Student *t*-test was performed. For the comparison of more than two groups, a one sample *t*-test was performed with a mu of 1 followed by the Bonferroni method of multiple hypothesis testing correction. Differences were considered statistically significant, indicated with stars, if the *P*-value was <0.05.

## RESULTS

### Establishing a quantitative assay for MicroDNA levels

**Figure 1.**
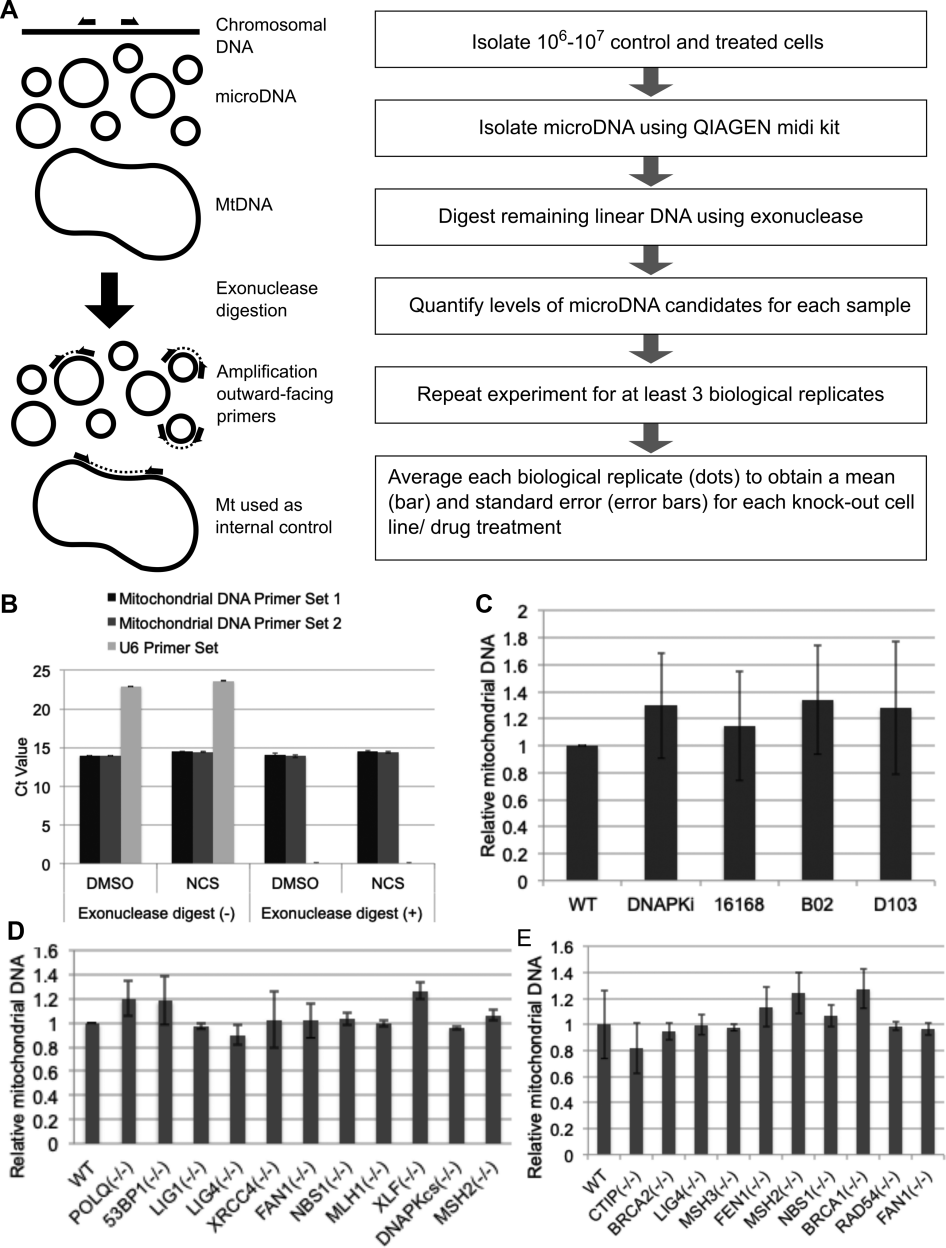
Assay developed to quantify microDNA. (**A**) Diagram of assay. (**B**) Linear DNA (U6 gene) is degraded by the exonuclease treatment and becomes undetectable in assay while the mitochondrial DNA survive. (C–E) Mitochondrial DNA that is used as normalizing factor does not vary when cells are treated with (**C**) small molecule inhibitors, or have specific DNA repair genes knocked out by the CRISPR/CAS9 system in (**D**) U2OS or (**E**) by HR in DT40 cells.

To determine the changes in eccDNA abundance under different conditions, we developed a quantitative assay that detects the levels of specific candidate sequences that represent the most abundant microDNA molecules detected by next-generation sequencing of microDNA in our previous RCA libraries (Figure 1A, Supplemental Table S1; NCBI GEO, GSE68644 and GSE36088). We took care to ensure that the distribution of properties of the assayed sites (Supplemental Table S3), such as GC content, genic location, derivation from unique sequences and flanking microhomology, were similar to the general properties of the hundreds of thousands of microDNA reported in our previous papers from high throughput sequencing of microDNA-derived RCA libraries (4,15).

The assay uses outward facing primers to selectively amplify circular microDNA across the circularizing junction (Figure 1A). The PCR primers were selected such that they had comparable efficiency of amplification and produced amplicons of comparable size (Supplemental Figure S1B, C and Table S1). We sequenced the amplicons to ensure that the inverse PCR was amplifying the correct sequence across the junction of the targeted circle (Supplemental Table S1).

To test whether the mutations or experimental treatments altered the general properties of the microDNA, we performed high-throughput sequencing and analysis of RCA libraries. The results show that the microDNA obtained from different mutant cell lines and after different chemical treatments generally have characteristics common among themselves and consistent with previously published microDNA, e.g. GC content, presence of microhomology at the ligation junction (Supplemental Figure S1F–H). The length distribution of the microDNA in one library from the MLH mutant cells looked different, but this is probably a technical artifact due to library quality, because the length distribution in these cells reverted to the distribution seen in other cells when the library was prepared after NCS or PARP1i treatment (Supplemental Figure S1F). Overall the length distributions of the microDNA were unchanged by the various mutations or chemical treatments. We additionally found that, as before, microDNA arose specifically from some hotspot regions (Supplemental Figure S1I-J).

To eliminate any contaminating signal from chromosomal DNA, especially amplification of chromosomal DNA with tandem duplications that may give a confounding signal, the eccDNA was enriched using a circular DNA plasmid isolation kit and contaminating linear DNA removed by digestion with an exonuclease for two 24 hours digestions. We confirmed the efficacy of this exonuclease treatment by showing that signal from the U6 gene on the linear chromosomal DNA disappeared after the treatment (Figure 1B). We also showed that inverse PCR primers from chromosomal sites not known to produce microDNA will not produce detectable product on linear chromosomal DNA after the exonuclease digestion (Supplemental Figure S1D).

The amplification of the specific microDNA was quantified using QPCR and the signal was normalized against mitochondrial DNA, which as a circular DNA molecule is protected by DNase digestions. The complete list of abundance of each microDNA in each biological replicate is shown in Supplemental Table S2. Because of the stochastic nature of microDNA generation, there is variability in the abundance of a circle in one experimental determination. However, the collective measurement of the abundance of eight different microDNA in a given replicate, and the inclusion of multiple biological replicates allowed us to manage the variability and carefully compare the abundance of microDNA molecules as a class across different experimental conditions and cell lines. The abundance of the normalizing mitochondrial DNA was shown to be unaffected by the various small molecule inhibitor treatments (Figure 1C) and gene knockouts in DT40 and U2OS cells (Figure 1D, E) that were used to perturb microDNA levels.

### MicroDNA formation is induced by double strand breaks

**Figure 2.**
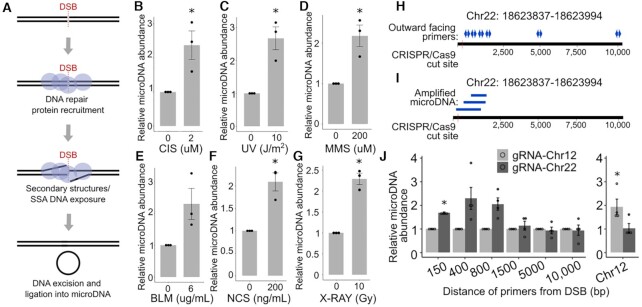
MicroDNA formation is induced by disruptions to DNA structure (**A**) Diagram of DSB leading to microDNA formation. (B–G) MicroDNA levels measured by the QPCR assay in 293T cells 48 h after the addition of (**B**) cisplatin, (**C**) UV, (**D**) MMS, (**E**) BLM, (**F**) NCS, (**G**) X-rays. Each dot is a mean of 8 eccDNA candidates within one biological replicate. Mean of three biological replicates ± S.E. indicated. (**H**) Diagram of induced DSBs at locus Chr22:18624104 in 293A cells and the amplification of circles arising proximal to the cut site using outward facing primers located as shown. (**I**) Diagram of microDNA sequences amplified by the outward facing primers confirmed by sequencing. (**J**) Quantification of microDNA isolated 48 hours after the transfection of an a p413 vector containing CAS9 and a gRNA sequence targeting Chr22:18624104 or Chr12:117100086. The locations of the microDNA measured are on the X-axis. Mean and S.E. of four biological replicates as indicated by four dots. *P*-values from one-sample t-tests <0.05 after correction for multiple hypothesis testing when necessary are indicated with a (*).

We hypothesized that microDNA formation was tied to DNA repair mechanisms, and thus microDNA levels would increase in a short time after DNA damage (Figure 2A). Treatment of 293T cells with various agents that disrupt the DNA structure, most notably, those that induce double-strand breaks (DSB), increased microDNA within 48 h (Figure 2B–G). Cisplatin, which leads to DNA crosslinking that is repaired by a pathway causing a DSB (18), increased the abundance of microDNA (Figure 2B). UV radiation induces thymine dimers and can also induce DSBs at high levels (18), and this increased microDNA levels (Figure 2C). MMS, which methylates DNA and causes DSB due to replication stalling (19), increases microDNA abundance (Figure 2D). Finally, two agents that directly cause DSB, Neocarzinostatin (NCS) and X-rays (20–22) increased microDNA abundance, while a third agent, Bleomycin increased the abundance though the result did not reach statistical significance (Figure 2E–G). Interestingly, the increase of microDNA by each type of damage plateaued around a 2-fold increase, suggesting that microDNA production is limited either by repair dynamics, or by the amount of damage that can be withstood by a cell.

### MicroDNA formation is increased at locus of DSB

Multiple DSBs on the same chromosome could release chromosomal fragments that are circularized to form eccDNAs. To determine whether a single DSB leads to the local induction of microDNA, we induced a DSB at a specific locus in 293A cells by CRISPR–Cas9 mediated targeting of a specific location in the genome (Chr22:18624104) (Supplemental Tables S4 and S5). The DSB cut site on Chr 22 was chosen because this region naturally forms low levels of microDNA which improves the sensitivity of the assay and contains unique sequences which can be targeted by PCR primers. Because transcription is known to influence eccDNA formation (23,24) we avoided genic sequences as well as sequences where GC content is significantly above or below the genome average. Finally, the sgRNA sequence at Chr 22 had >99.9% specificity to the targeted site and its cutting efficiency was >60% (Supplemental Figure S2). To ensure that the induction was specific to the area near the Chr 22 cut, we measured the microDNA from another microDNA hotspot on Chr 12 (Supplemental Tables S4 and S5).

The amount of microDNA produced from the Chr 22 locus was measured using outward-directed primer pairs located at distances of 150, 400, 800, 1500, 5000, 10 000 bp from the DSB site (Figure 2H, I). MicroDNA were stimulated proximal to the DSB: 150, 400, 800 bp away from DSB, though statistical significance was only reached with the primers 150 bp away from the DSB (Figure 2J, listed in Supplemental Table S4). MicroDNA further from the DSB were not increased. These results show that a DSB induces the formation of microDNA from the chromosomal DNA proximal to the DSB. In contrast, the Chr 12 locus did not increase microDNA production when cells were transfected with the Chr 22 sgRNA (Figure 2J) showing that the effect was local to the site of DSB cut. A similar locus-specific induction of microDNA was seen with an sgRNA targeting a microDNA hotspot on Chr 12 and primers ∼300 bp away from the cut site (Figure 2J, last pair of bars). Therefore, the induction of microDNA near a targeted single DSB was not specific just to the Chr 22 locus. Although differences in priming efficiency complicate our ability to quantitatively compare the abundance of microDNA from different loci, two of the microDNA produced from the site-specific DSB site on Chr 22 appear to be comparable in abundance to the endogenous microDNA from 6 of the 8 hotspot sites that were assayed in the other experiments (Supplemental Table S6).

Thus, the formation of the microDNA did not require two closely spaced DSB to release a chromosomal fragment that is circularized. Instead, they can be produced as a byproduct of repair of a single DSB.

### MicroDNA formation is suppressed by c-NHEJ

**Figure 3.**
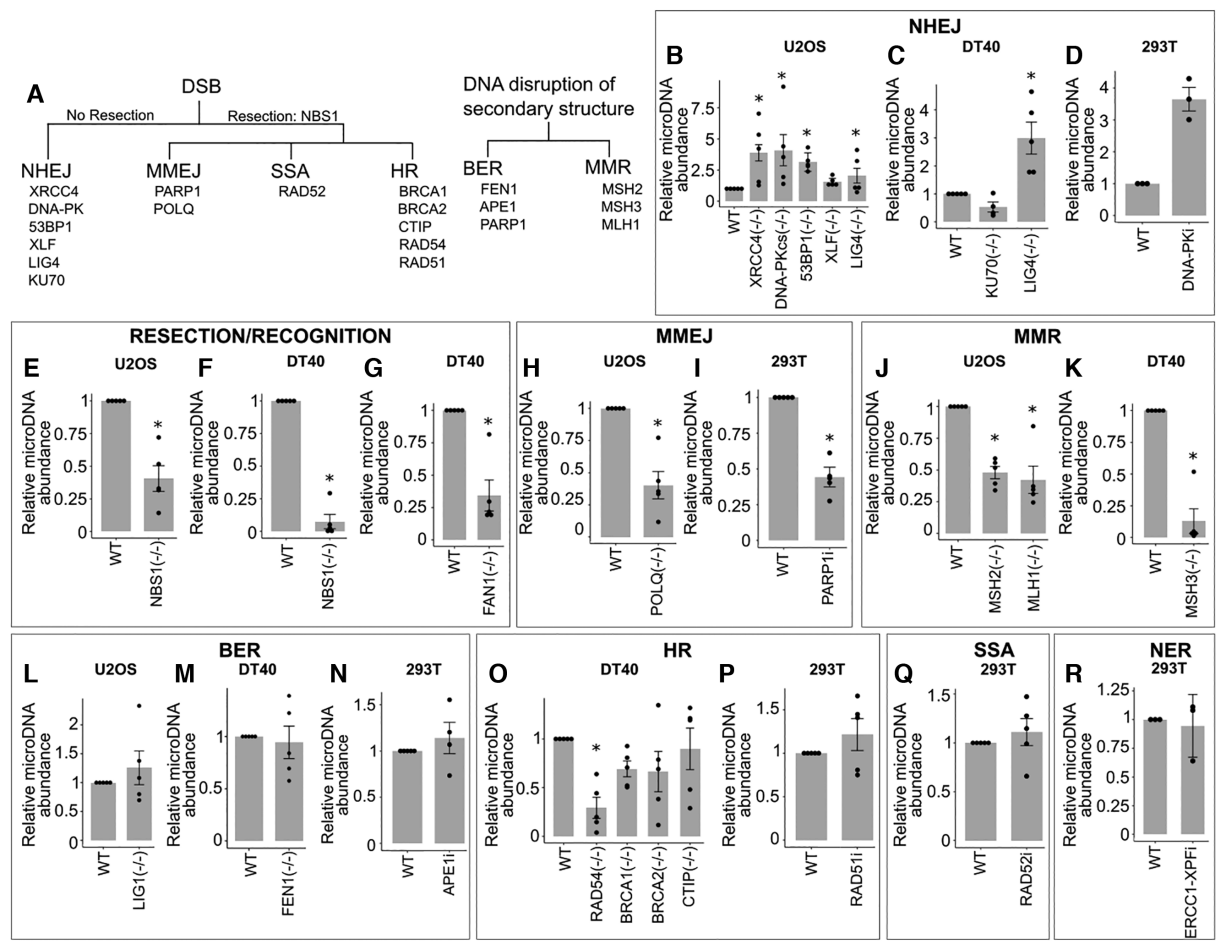
MicroDNA formation is suppressed by c-NHEJ and increased by resection based repair pathways (**A**–**E**) Levels of microDNA in U2OS cell lines with various knocked-out genes from DNA repair pathways. (A) Diagram of genes knocked-out or inhibited and pathways they are implicated in. Relative levels of microDNA in different knock-out and small molecule inhibitor of DNA repair pathways: (B–D) NHEJ, (**E**–**G**) resection and recognition of DSBs, (**H**–**I**) MMEJ, (**J**–**K**) MMR, (**L**–**N**) BER, (**O**–**P**) HR, (**Q**) SSA, (**R**) NER. *P*-values from one-sample *t*-tests <0.05 are indicated with a (*), and in panels where multiple conditions are tested, we corrected for multiple hypothesis testing. Each dot is a mean of eight eccDNA candidates within one biological replicate. Mean of five biological replicates ± S.E. indicated.

**Figure 4.**
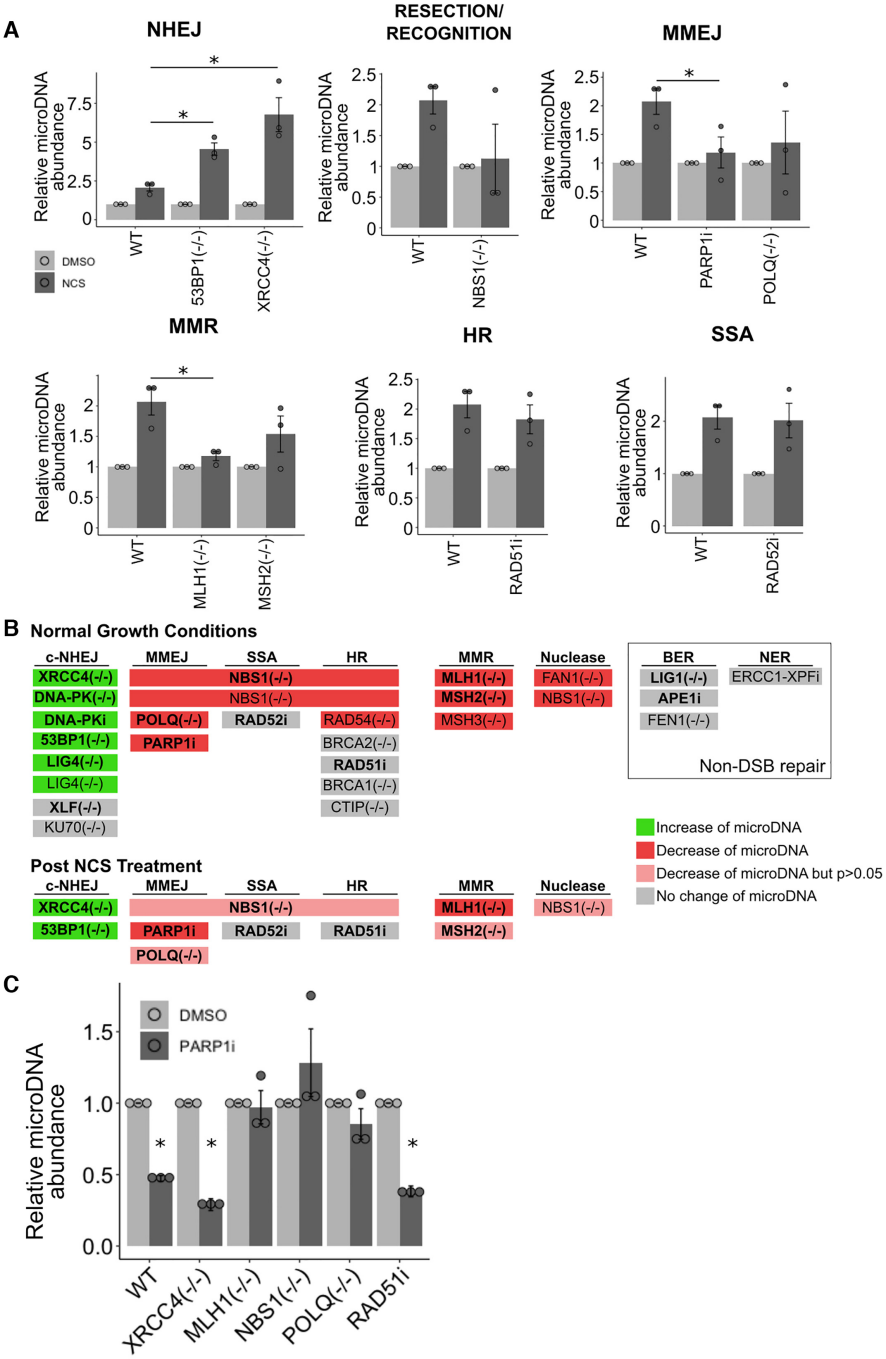
After DSB, cells lacking c-NHEJ have more microDNA and cells lacking functional MMEJ and resection proteins have fewer microDNA. (**A**) Levels of microDNA after treatment of NCS (200 ng/ml) for 48 h. *P*-values < 0.05 in a two-sample t-test (relative to abundance in NCS treated WT cells) with correction for multiple hypothesis testing when needed are indicated with a (*). (**B**) Graphical summary of whether mutants or inhibitors of a given repair pathway changed the microDNA abundance: increase (green), decrease (red) or decrease but did not reach statistical significance (pink). (**C**) Levels of microDNA after treatment of AZD2461 (10 uM) for 48 h. *P*-values <0.05 in a one-sample t-test (relative to DMSO treated cells) followed by multiple hypothesis testing correction are indicated with a (*). Mean ± S.E. of three biological replicates shown.

To elucidate which DNA repair pathways form microDNA as products of repair, we utilized our quantitative assay for microDNA on an array of isogenic cell lines that have DNA repair genes knocked-out with CRISPR or inhibited by specific molecular inhibitors (Figure 3A–R). The results from human and chicken cells under normal growth conditions are summarized in Figure 4C Human U2OS cells that lacked functional genes in the c-NHEJ pathway, *XRCC4*, *DNA-PKcs* and *LIG4*, all had a significant increase of microDNA; *53BP1*, while not being a primary player in c-NHEJ, favors c-NHEJ by preventing hyper-resection at DSB ends, and loss of 53BP1 also increases microDNA levels (Figure 3B–D). XLF was the only exception whose loss did not increase microDNA abundance. The critical role of DNA-PK in suppressing microDNA production is supported by the increase of microDNA in human embryonic kidney derived 293T cells treated with the specific DNA-PK inhibitor (CAS-20357-25-9), though the *P*-value of 0.07 missed statistical significance (Figure 3D).

We later show that cells in S phase contain more microDNA. It is important to note that the cells mutated in c-NHEJ pathway above do not have a significant increase in S phase cells (Supplementary Figure S4B, C). We also tested that for at least one of the c-NHEJ mutant cells, *XRCC4*–/–, there is no global increase in DNA damage as detected by γH2AX Westerns (Supplementary Figure S5B). Together, these data suggest that c-NHEJ suppresses microDNA formation, and that microDNA levels rise significantly when c-NHEJ is compromised.

To determine how general this result is, we used chicken DT40 lymphoma cells knocked out for various DNA repair genes. Lack of *LIG4*, important for c-NHEJ, in DT40 cells also increased the microDNA levels (Figure 3C). The knock-out of *KU70* did not change microDNA levels significantly, but we suspect that this could be explained by the higher levels of cell-death experienced in this cell line. KU70 is known to be an essential gene in mammalian cells (25), so that it is likely that the *KU70–/–* DT40 cells adapted to the loss of KU70 in some way that alters normal NHEJ DNA repair. Broadly, these results show that the formation of microDNA is suppressed by the repair of the DSBs by the c-NHEJ pathway.

### MicroDNA formation is facilitated by proteins required for MMEJ

We next tested microDNA abundance in resection-based DNA repair pathways which require resection to rejoin DNA strands after damage, including MMEJ, SSA, and HR. NBS1, as a component of the MRN complex, is recruited early to DSB, and is critical for resecting DNA ends and a major factor in repair choice from c-NHEJ to end-resection dependent SSA and HR repair (26). The knock-out of *NBS1* significantly decreased microDNA abundance both in human U2OS and in chicken DT40 cells (Figure 3E, F). Further, loss of *FAN1* a nuclease implicated in interstrand cross-link repair and replication fork stability (27,28), also significantly reduced microDNA levels (Figure 3G). Together these data suggest that nucleases involved in DNA resection promote microDNAs formation.

We next turned to the contribution of proteins within the MMEJ DNA repair pathway, which requires a small degree of resection and homology for repair (29). Loss of *POLQ*, a helicase-polymerase involved in unwinding DNA and facilitating the annealing of homologous ssDNA in MMEJ (30)(30), significantly reduced levels of microDNA (Figure 3H). PARP1 tethers DNA ends and interacts with XRCC1 and LIGIII to promote MMEJ (31), and the lack of PARP1 greatly reduces MMEJ (29). Addition of a PARP1 inhibitor, AZD2461, to 293T cells reduced levels of microDNA (Figure 3I). The reduction in microDNA upon inhibition of PARP1 and POLQ suggests that MMEJ is a major DNA repair pathway contributing to the observed endogenous levels of microDNA.

The MMR pathway is known to have some interaction with MMEJ (32,33). We therefore tested whether the loss of proteins within MMR would alter eccDNA abundance. Knock-out of *MSH2* or *MLH1* in U2OS cells, as well as knock-out of *MSH3* in DT40 cells, led to a substantial decrease of microDNA (Figure 3J, K). Combined, these data show that end-resection, MMEJ, and MMR all contribute to microDNA formation.

Here again, we took the precaution of examining the cell-cycle profile and γH2AX levels of selected mutants in U2OS to ensure that there was no global change in S phase population or basal level of DNA damage to indirectly influence the microDNA abundance (Supplementary Figures S4B, C, S5B).

### MicroDNA formation independent of BER

PARP1 inhibition is known to disrupt base excision repair (BER) of some types of DNA lesions (34). To determine whether the PARP1 inhibitor is altering microDNA levels through the BER pathway, we tested microDNA levels after the inhibition of two genes within the BER pathway. The knock-out of LIG1, the ligase utilized by BER (35), did not significantly change microDNA abundance in U2OS cells (Figure 3L). Furthermore, the knock-out of FEN1, the endonuclease known to be necessary for BER by processing flap-containing intermediates (36), did not change microDNA abundance in DT40 cells (Figure 3M). The APE1 endonuclease is necessary at an early stage in BER because it is recruited to the apurinic sites to cut the DNA and recruit other BER proteins (37). Consistent with the lack of an effect of BER on microDNA production, an APE1 inhibitor (Mx) did not significantly alter eccDNA abundance in 293T cells (Figure 3N). Therefore, the role of PARP1 in promoting microDNA production we noted in Figure 3I is likely dependent on its role in MMEJ but not in BER. (Effectiveness of chemical inhibitors verified in Supplemental Figure S3).

### Endogenous MicroDNA formation is independent of the HR pathway

To test the contribution of HR to microDNA formation, we tested cells lacking HR genes including *RAD54, BRCA1, BRCA2* or *CTIP* in DT40 cells (Figure 3O). Mixed effects were seen. *RAD54* loss significantly decreased microDNA levels. In contrast, *BRCA1*, *BRCA2* or *CTIP* loss did not significantly decrease microDNA levels. A RAD51 inhibitor on 293T cells (B02: Figure 3P) also did not decrease microDNA levels. The requirement of NBS1 from the MRN nuclease complex, but not of CTIP could be explained by the fact that the MRN complex has an endonuclease activity that is essential for end resection, that MRN can bind and resect DNA slowly but independently of CTIP and BRCA1 (38), or that other enzymes like DNA2 and EXO1 are also involved in strand resection with CTIP but after MRN action (39). The unique requirement of *RAD54* and none of the other HR factors suggests that the effect seen with *RAD54* loss is due to its role in pathways not involving HR. *RAD54* is known to be involved in resolving looped DNA structures, not only in strand invasion but in combination with *MUS81*-*EME1* to resolve various DNA structures (40–43). RAD54 may be the necessary component to release the looped DNA structures, e.g. a D-loop formed by annealing of microhomology sites while forming the eccDNA (Figure 6).

### MicroDNA formation independent of SSA and NER

To test other repair pathways downstream from MRN-mediated end-resection, we analyzed microDNA levels in cells lacking functional proteins for single strand annealing (SSA) repair. RAD52 is necessary for SSA because it mediates strand invasion in a RAD51 independent manner (44). We found no change in microDNA abundance after the addition of the RAD52 inhibitor, D-103, to 293T cells (Figure 3Q), suggesting that SSA is not essential for microDNA formation. Further, the production of microDNA is not dependent on the NER pathway, as tested with the inhibition of ERCC1-XPF with NSC16868 (45,46) (Figure 3R).

### Cells lacking c-NHEJ produce more microDNA after DSBs

To examine the interaction between DSBs and the repair pathways implicated above in microDNA formation, we induced DSBs in cells with compromised DSB repair by the addition of NCS (Figure 4A, B). Cells lacking genes that promote the c-NHEJ, *53BP1 and XRCC4*, produced more microDNA upon addition of NCS compared to WT cells (Figure 4A). This result is consistent with results above suggesting that c-NHEJ repair decreases endogenous microDNA levels.

Cells compromised in resection-based repair pathways fail to increase microDNA levels after DSB induction. The loss of *NBS1* (resection), inhibition of PARP1 and loss of *POLQ* (MMEJ) decrease microDNA abundance after NCS treatment relative to the level seen in WT cells (Figure 4A), though only the effect of PARP1i reached statistical significance. *MLH1* and *MSH2* (MMR) loss suppressed the increase of microDNA after DSBs (Figure 4A), though the abundance after *MSH2* loss was not statistically significantly different from that seen in WT cells after correction for multiple hypothesis testing. These results are consistent with the hypothesis that resection, MMEJ and MMR are important for microDNA production both under basal conditions as well as following the induction of DSBs by DSB-inducing agents.

The RAD51 inhibitor (HR) or the RAD52 inhibitor (SSA) did not significantly alter the microDNA abundance post NCS (Figure 4A). Overall this supports the hypothesis that as in basal conditions, microDNA formation after DSB is not dependent on HR or SSA.

γH2AX western after DSB induction in the *XRCC4–/–*, *NBS1–/–* and *MLH1–/–* cells showed the extent of damage is comparable to that in WT cells, and so differences in the damage level do not explain the increase (*XRCC4–/–*) or decrease (*NBS1–/–* and *MLH1-/-*) in microDNA induction in these mutant cells post DSB. The *POLQ–/–* cells showed an increase in γH2AX signal, which also suggests that a decrease in DNA damage cannot explain the decrease in microDNA in these mutant cells.

Further, the addition of PARP1i decreased microDNA formation in cells lacking effective c-NHEJ (*XRCC4–/–*) or HR (RAD51i) pathways (Figure 4C), suggesting that MMEJ contributes to the increase of microDNA seen in cells compromised for c-NHEJ repair. As expected, the PARP1i did not further decrease the microDNA levels in cells lacking effective MMEJ (*POLQ*–/– and *NBS1–/–*), consistent with the idea that the PARP1i decreases microDNA levels by impairing MMEJ. Interesting, PARP1i also did not decrease microDNA levels in cells mutated in MMR (*MLH1–/–*) pathways, suggesting that the MMR genes interact with MMEJ to contribute to microDNA levels.

### MicroDNA formation is increased in S-, G2- and M-Phase of the cell cycle

**Figure 5.**
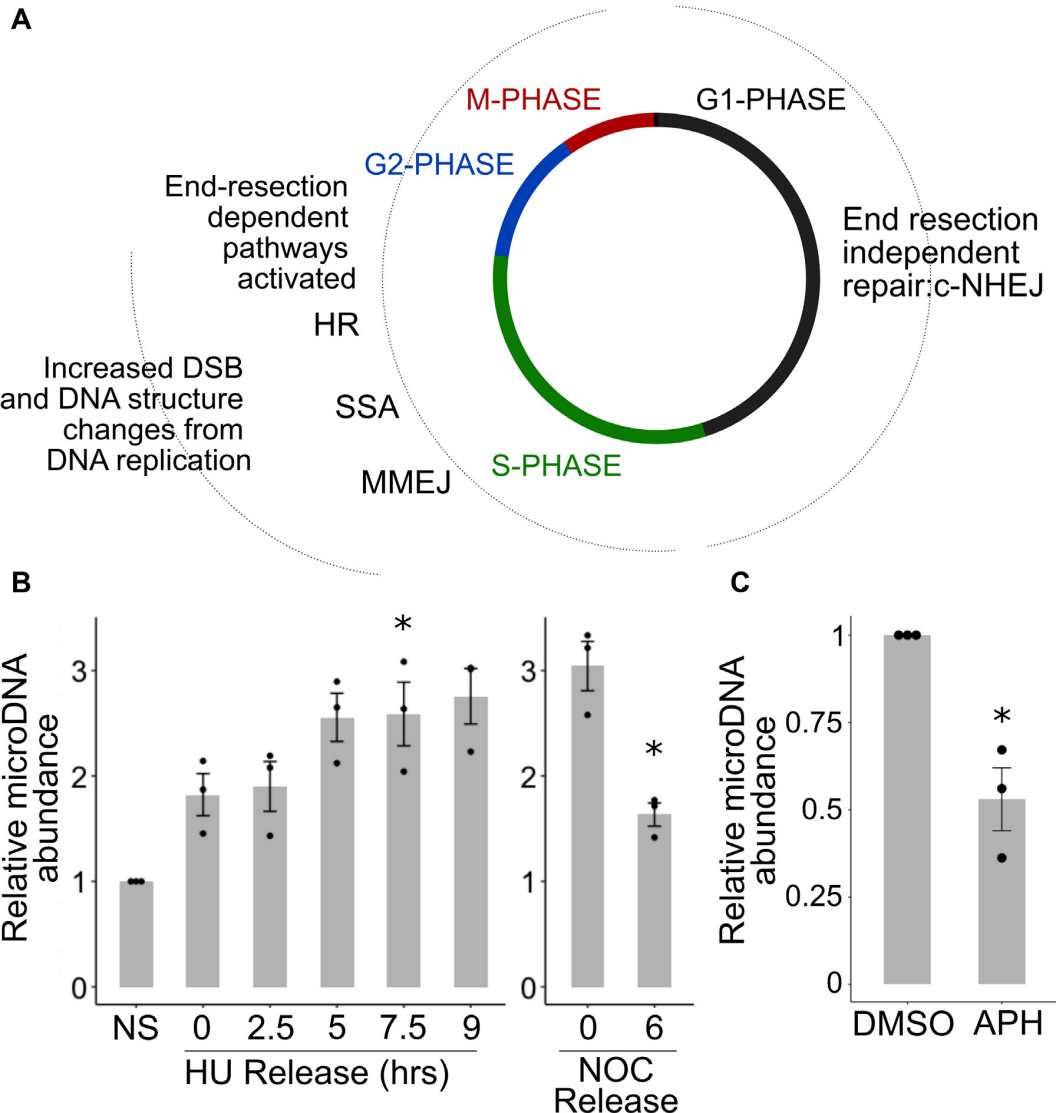
MicroDNA formation is increased in S-phase, G2-phase, and M-phase of the cell cycle. (**A**) End-resection-independent pathway of repair is active in G1, while end-resection (and homology) dependent repair is more active in S and G2. (**B**) Left: microDNA levels in S-phase, Right: MicroDNA levels as cells pass through M-phase to G1. *P*-values <0.05 in a one-sample *t*-test, corrected for multiple hypothesis testing for the left panel, are indicated with a (*). (**C**) MicroDNA levels after the addition of 3 uM aphidicolin (APH). *P*-value <0.05 in a one-sample t-test is indicated with a (*). Mean ± S.E. shown

The extent of endogenous DSBs and the utilization of different DNA repair pathways are different in different parts of the cell cycle (Figure 5A, B). DSBs are increased during normal DNA replication in S phase when the replication fork runs into nicks or other barriers to DNA replication (47). Further, c-NHEJ is used throughout the cell cycle, but repair pathways dependent on end-resection are used only in S- and G2-phase (25). Therefore, we hypothesized an increase of eccDNA through S-phase.

Cells blocked at the G1-S phase transition in hydroxyurea were released from the block by washing the cells, and cells harvested at 2.5, 5, 7.5 and 9 h after release. FACS for analysis confirmed that the cells progressed through S-phase (Supplemental Figure S4A). The microDNA levels were increased by the HU block and increased further as the cells progressed through S-phase, though the induction relative to non-synchronized cells was statistically significant only in the 7.5 h time point after correction for multiple hypothesis testing (Figure 5B). To ensure that the increase was not due to DNA damage specifically induced by hydroxyurea, we repeated the experiment following thymidine block and release and observed the same trend of increase in microDNA levels as cells progressed through S phase (Supplementary Figure S4D, E). Finally, we measured mitochondrial DNA levels as cells pass through S phase to rule out the possibility that a decrease in the normalizing mitochondrial DNA levels accounts for the observed increase in microDNA levels in S phase (Supplementary Figure S4F). These results suggest that either the increase in DNA damage in S phase, or the induction of DNA repair pathways to repair DSB or resolve stalled or collapsed forks in S phase, contribute to the formation of microDNA.

We tested the levels of microDNA in cells blocked in M-phase by nocodazole. The levels of microDNA were elevated three-fold relative to non-synchronous cells (Figure 5B) but the extent of elevation may be a slight under-estimate because the normalizing mitochondrial DNA level was also increased about 1.5-fold (Supplementary Figure S4F). When we released the cells from M-phase for 6 h to allow them to progress into G1, the microDNA levels declined significantly (Figure 5B). This suggests that the microDNA may be exposed to the cytoplasm during cell division and experience some degradation.

Together, this shows that microDNA levels increase during S-phase when DSBs appear and resection-dependent DNA repair pathways are most active. Consistent with a role of DNA replication related DSBs in producing microDNA, prevention of DNA replication with aphidicolin, an inhibitor of replicative DNA polymerases, lowers microDNA levels (Figure 5C).

## DISCUSSION

**Figure 6.**
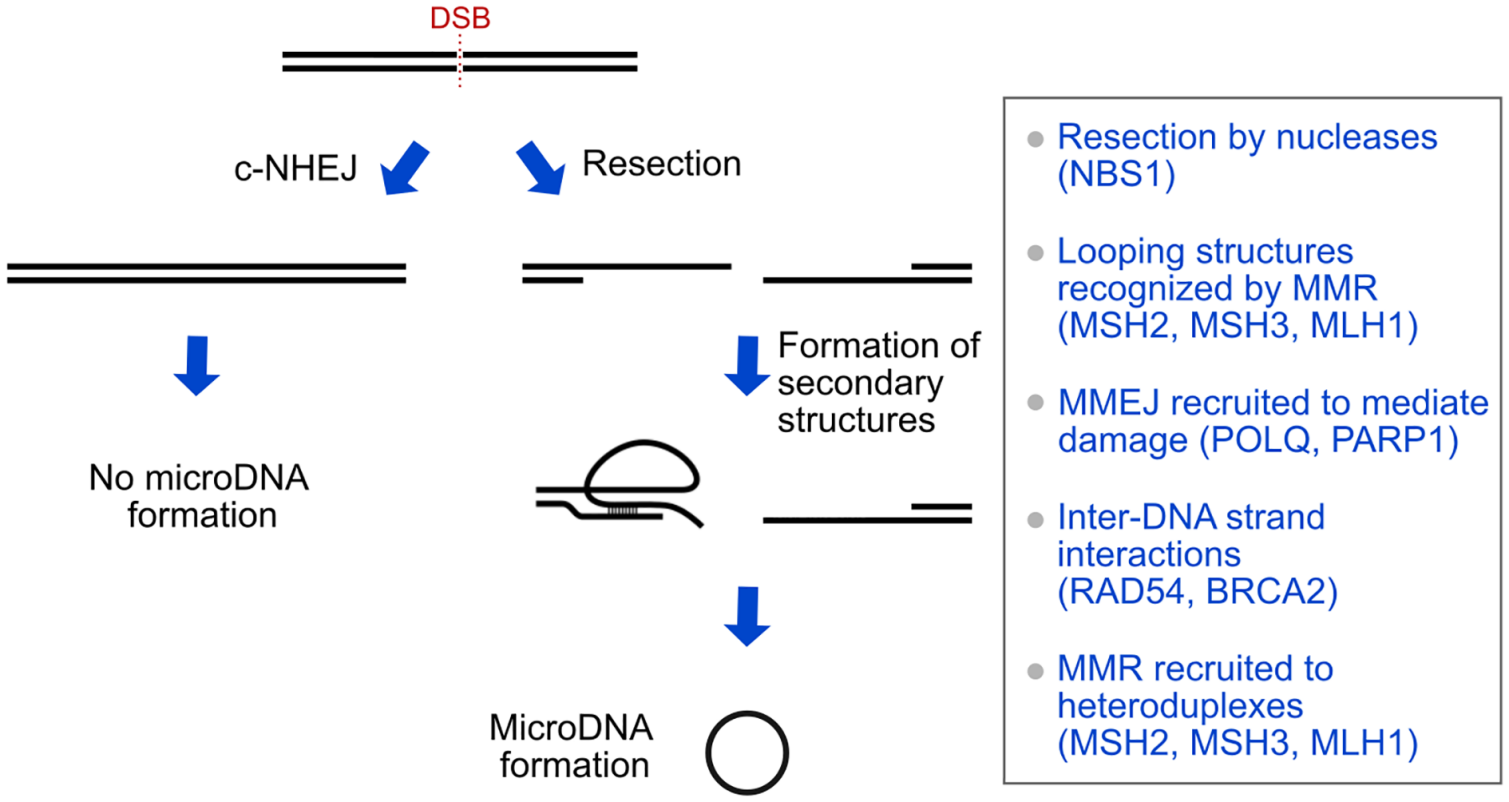
Model of endogenous microDNA formation from a single DSB. MicroDNA are stimulated by DSB repair that involves end-resection and their production likely involves the resolution of secondary structures caused by microhomology on end-resected DNA during the DNA repair.

MicroDNA are formed by DNA repair pathways, especially after DSB and during replication (Figure 6). The DNA repair proteins that are necessary for microDNA after DNA damage are mostly tied to MMEJ, DNA end-resection and homology searching, i.e. POLQ, PARP1, MSH2, MSH3, MSH6, MLH1, NBS1, FAN1 and RAD54. Additionally, proteins that favor c-NHEJ, which suppresses resection based DSB repair, suppress the formation of microDNA, i.e. XRCC4, DNA-PKcs, LIG4 and 53BP1. Together this shows that following even a single DSB, DNA repair pathways which resect the DNA and produce a single-strand tail promote microDNA formation. We hypothesize that the single-stranded DNA uses microhomology to form secondary structures with the double stranded DNA in *cis*, and this leads to aberrant DNA structures which are cleaved from the chromosome to form microDNA (Figure 6). Blocking c-NHEJ shunts the DSB to resection-mediated repair, which leads to an increase in microDNA.

The genetic information excised during the process could be restored by HR repair from the sister chromatid, so that microDNA formation is not accompanied by extensive chromosomal deletions. This is consistent with our previous observation that somatically mosaic chromosomal micro-deletions are rare even at hotspots of microDNA formation (4). We also confirmed experimentally by PCR at three hotspots of microDNA formation that the *XRCC4* mutation-mediated increase in microDNA is not accompanied by microdeletions at the genomic locus (Supplemental Figure S1E). That microDNA formation is mostly not coming from deletion of genomic sequence is also consistent with a recent study on eccDNA containing the HXT6/7 gene under selection in yeast cells (24).

We speculate that MMR may be involved in microDNA formation because the MMR genes have a role in recognizing and repairing mismatches and looping structures that occur during annealing of short homologous sequences (32,48).

A limitation of our study is that we have not been able to consistently examine exactly the same genes in human U2OS cells and in chicken DT40 cells. We were limited by the cell lines that were already available in DT40 (where they have been made over decades using homologous recombination), and the knockout cell lines we managed to obtain in U2OS using CRISPR-Cas9. Thus, sometimes we had to use deletions in different genes in the same pathway, and hence our conclusions are more about pathways than about specific genes. There are also cell-line-specific differences that make it difficult to obtain the same gene knockouts in chicken DT40 and human U2OS cells.

Another limitation of the study is that we have measured the levels of microDNA after various perturbations, and not the actual rates of formation or degradation. Theoretically, it is possible that the differences we observe are due to differences in degradation of the microDNA. However, there is nothing in the Literature to suggest that mutations in c-NHEJ or MMEJ pathways will systematically alter the degradation of circular DNA. Furthermore, the rapid increase in microDNA levels in 3 hours following release from a hydroxyurea block is also consistent with hypothesis that most of these changes in microDNA levels are due to changes in the formation of the microDNA.

The increase of microDNA in hydroxyurea and thymidine blocked cells (when the cells have replication stress), and during S phase, suggests that endogenous microDNA formation is connected to repair pathways associated with fork stalling. The decrease of microDNA as cells pass through mitosis into G1 suggests that the microDNA experiences some degradation in dividing cells.

The cell-cycle and DNA damage dependent changes in microDNA levels prompted us to ensure that the changes in microDNA abundance in the various mutant cell lines were not due to changes in the cell-cycle or the endogenous DNA damage levels in these cells. Cell-cycle analysis (Supplemental Figure S4) and gamma H2AX westerns (Supplemental Figure S5) of key cell lines indicate that this is not the case.

It has been previously shown that the induction of two DNA DSBs induced by exogenous CRISPR/Cas9 induction within the same chromosome can lead to eccDNA formation from the excised DNA by c-NHEJ (49). These findings suggest that c-NHEJ can contribute to eccDNA formation, but only when there are two DSBs on the same chromosome. Such concordant DSBs *in cis* are unlikely to be common in normal cells, because that would lead to frequent chromosomal deletions, and so the endogenous microDNA are most likely formed from single DSBs. However, this does not rule out eccDNA formation accompanied by chromosomal deletions in other contexts, e.g. when forming the large multi-locular circles called double-minutes or ecDNA in cancer cells undergoing chromothripsis.

Recently, it has been shown in yeast that endogenous eccDNA formation is tied to SAE2 and MRE11, proteins that resect DNA in DSB repair (24). MRE11 of the MRN complex is important for eccDNA formation in yeast (24). consistent with our finding that NBS1 of the MRN complex is important for microDNA formation in human and chicken cells. We could not use an MRE11 inhibitor Mirin to directly test the role of MRE11 in forming endogenous eccDNAs because of the very high toxicity of the drug in 48 h, the minimal period at which we measured microDNA levels (Supplemental Figure S3). MUS81 nuclease, which when paired with EME1 is involved in the unhooking of an interstrand cross-link, is also required for eccDNA formation in yeast (42). This is similar to the requirement we note for FAN1, though it is possible that MUS81 in yeast and FAN1 in human cells are required not to cut near a crosslink, but to cut a flap to release a circle in the model in Figure 6.

The increase in microDNA abundance following DSB could be relevant to the use of chemotherapeutic agents or radiotherapy, which lead to DSBs, and may, therefore, increase somatically mosaic microDNA, thus increasing the genetic variation of cancer cells and potentially leading to adaptation of the cancer to therapy. We show that highly abundant microDNA in NCS treated cells contain exonic and promoter sequences (Supplemental Figure S1I). which may affect cellular gene expression as demonstrated in eccDNA literature (5,50,51). Compromise of the c-NHEJ pathway will lead to increased abundance of microDNA. This could be relevant to cancers where c-NHEJ is known to be down-regulated in cancers such as breast cancer (52) as it suggests that the loss of c-NHEJ can increase genomic instability and tumor adaptation through increased formation of somatically mosaic eccDNA. Conversely microDNA formation can be decreased by inhibiting the MMEJ pathway. Because the MMEJ pathway is considered a non-essential pathway for normal cells (29), targeting this pathway would be a specific and non-toxic therapeutic option, if the monolocular circle population contributes to oncogene amplification or if MMEJ is used to join the fragments in multilocular double minutes.

## Supplementary Material

gkab984_Supplemental_File

## Notes

Present address: Anindya Dutta, Department of Genetics, University of Alabama, Birmingham, AL 35294-0024, USA.
