# Supplementary material for: MicroDNA levels are dependent on MMEJ, repressed by c-NHEJ pathway, and stimulated by DNA damage
Source: Nucleic Acids Res. 2021 Oct 30;49(20):11787–99. doi: 10.1093/nar/gkab984 (PMC8599734; doi:10.1093/nar/gkab984)
Supplement: gkab984_Supplemental_File [file gkab984_supplemental_file.pdf]

SUPPLEMENTAL MATERIALS

Supplemental Figure 1:

Outward facing primers target the known junction sequence of the microDNA and will therefore specifically amplify the sequence only if it has become circularized. (**A**) The primers are designed to create amplicons of similar size even though the microDNA targeted by the primers range from 180-1300 base pairs. (**B** and **C**) The outward facing primers measuring the eight microDNA (U2OS cells) are approximately equal in efficiency at detecting fold change in microDNA abundance. (**D**) Outward facing primers do not amplify sequences of regions known to contain no eccDNA by NGS. (**E**) Chromosomal loci that produce eccDNA do not experience detectable deletions by PCR even in XRRC4 mutant cells that have elevated microDNA production. (**F-H**) Sequencing data. Characteristics of microDNA from the U2OS knock-out cell lines: (**F**) Length distribution, (**G**) GC content, and (**H**) microhomology. (**I**) Highly abundant microDNA in U2OS cells after treatment of DSB inducing agent (NCS) containing promoter or exonic sequences. (**J**) MicroDNA arise from specific hotspot regions within genes.

(**A**)

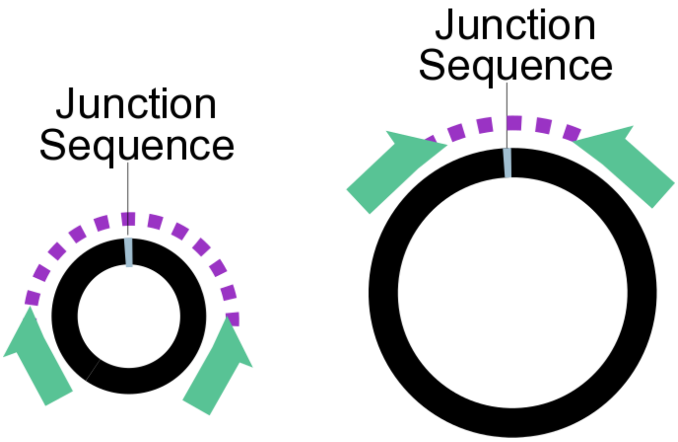

(**B**)

Dilution	Log(Quantity)	EccDNA 1	EccDNA 2	EccDNA 3	EccDNA 4	EccDNA 5	EccDNA 6	EccDNA 7	EccDNA 8
1	0	14.9	18.0	31.9	19.3	19.0	19.1	28.4	29.4
0.5	-0.301	16.0	19.1	33.0	20.6	20.1	20.3	29.6	30.5
0.25	-0.602	17.1	20.3	34.1	21.6	21.2	21.4	30.7	31.6
	**SLOPE**	-3.69	-3.82	-3.73	-3.84	-3.60	-3.79	-3.88	-3.74
	**EFFICIENCY**	**86.5**	**82.8**	**85.3**	**82.3**	**89.4**	**83.5**	**80.9**	**85.0**

(**C**)

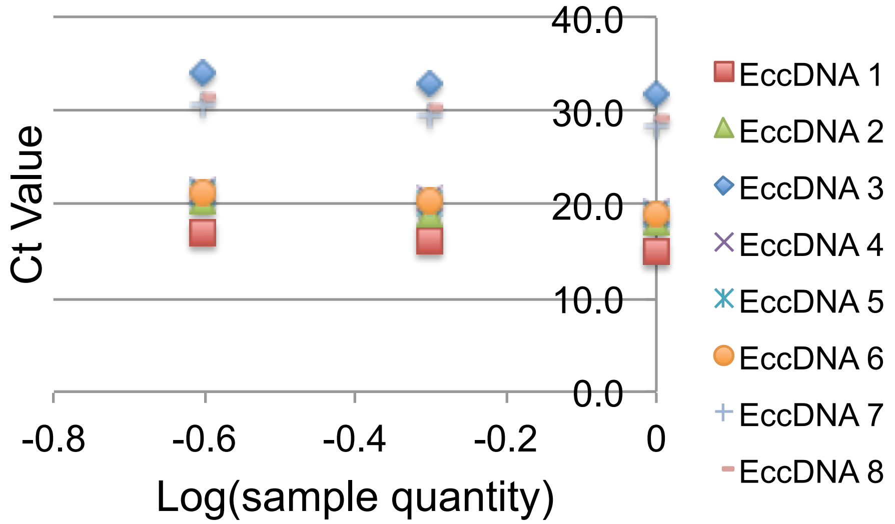
Ct Value
40.0
30.0
20.0
10.0
0.0
-0.8
-0.6
-0.4
-0.2
0
Log(sample quantity)
EccDNA 1
EccDNA 2
EccDNA 3
EccDNA 4
EccDNA 5
EccDNA 6
EccDNA 7
EccDNA 8

(**D**)

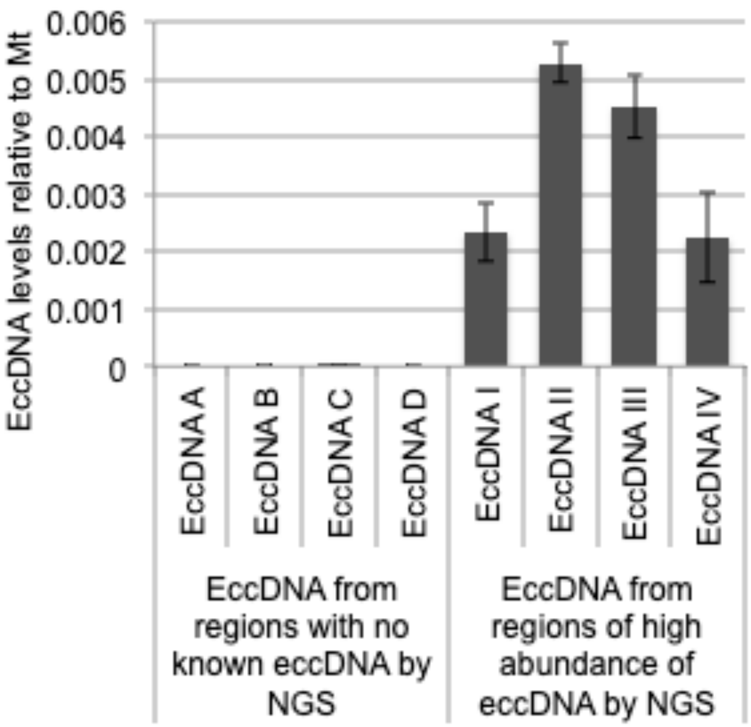
EccDNA levels relative to Mt
0.006
0.005
0.004
0.003
0.002
0.001
0
EccDNA A
EccDNA B
EccDNA C
EccDNA D
EccDNA I
EccDNA II
EccDNA III
EccDNA IV
EccDNA from regions with no known eccDNA by NGS
EccDNA from regions of high abundance of eccDNA by NGS

(**E**)

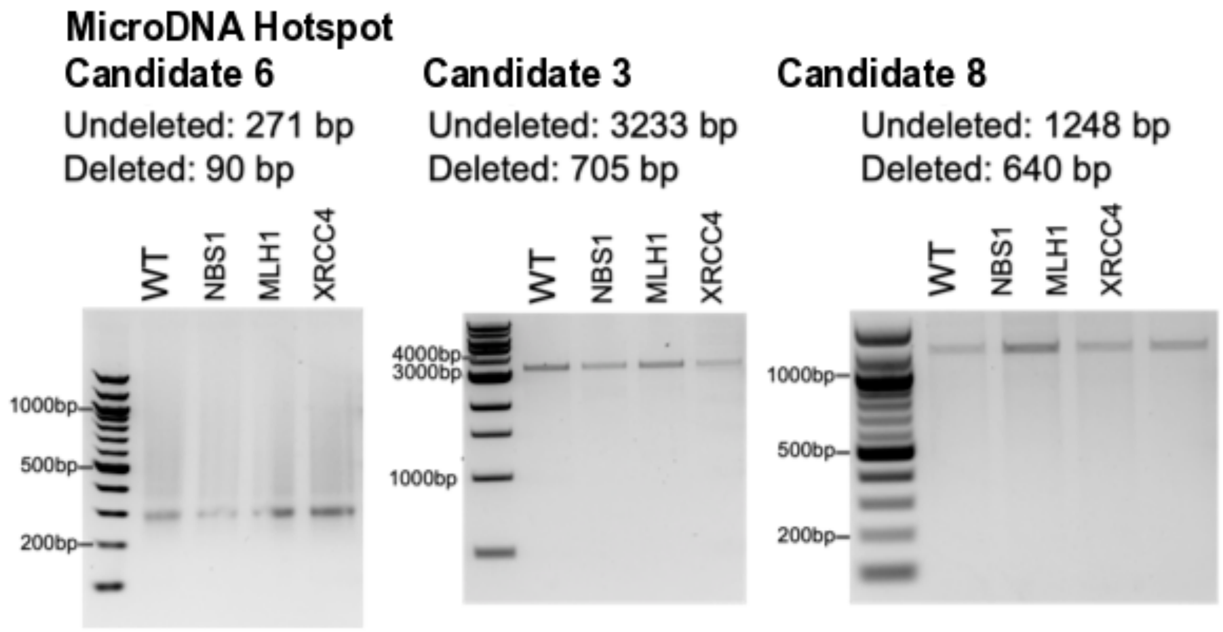

(**F**) Length Distribution of microDNA identified by sequencing RCA products from U2OS cells, WT, mutant in indicated genes, and treated with NCS or PARP1inhibitor. The length distributions are unchanged by the mutations or the treatment with chemicals.

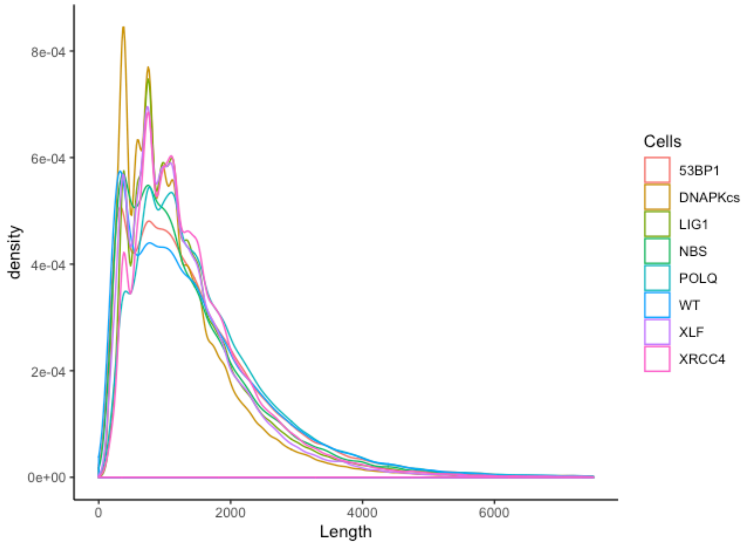

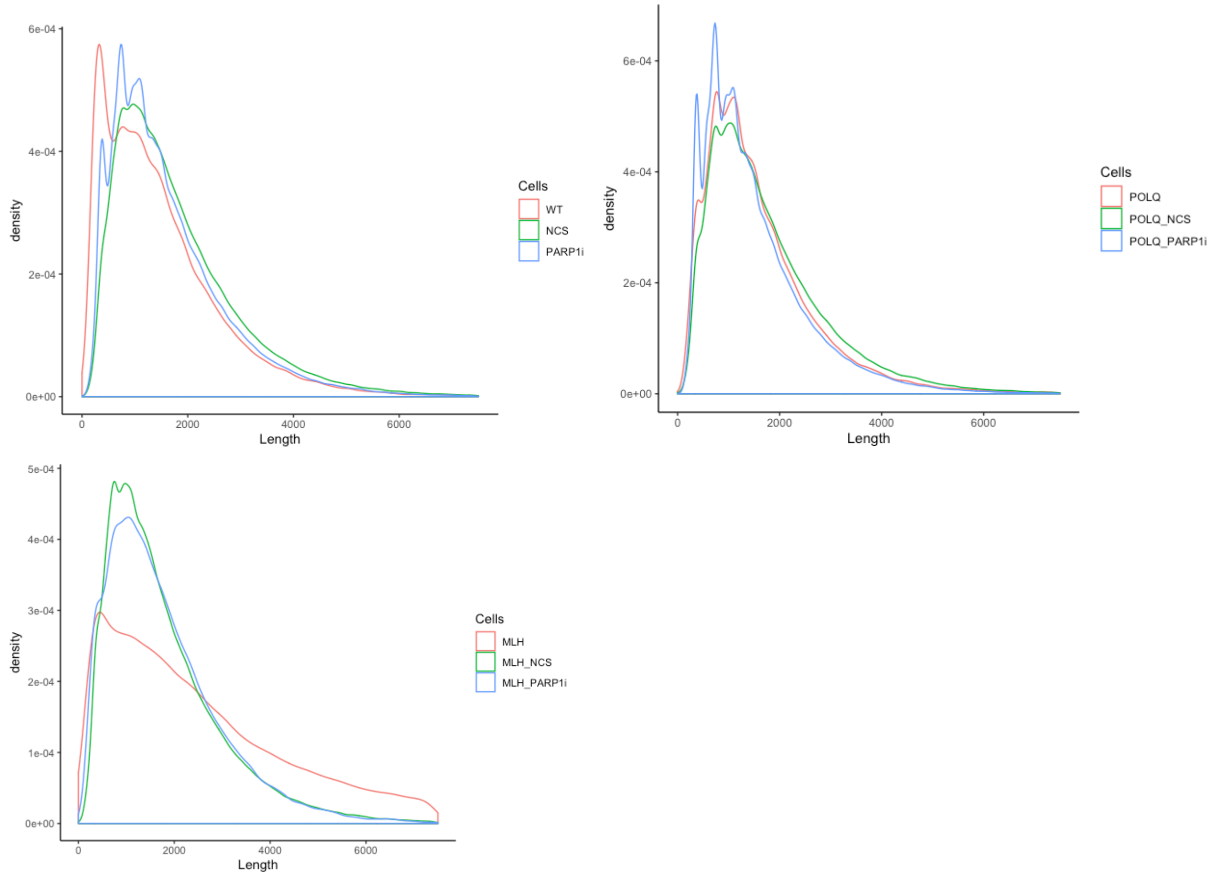

(**G**) GC content of the microDNA are unchanged in the various libraries prepared from U2OS cells as in (F).

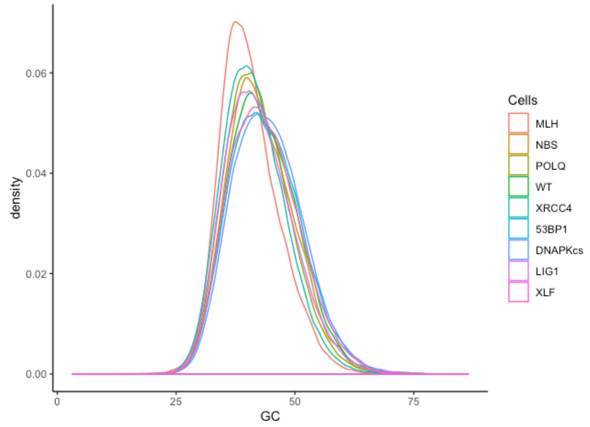

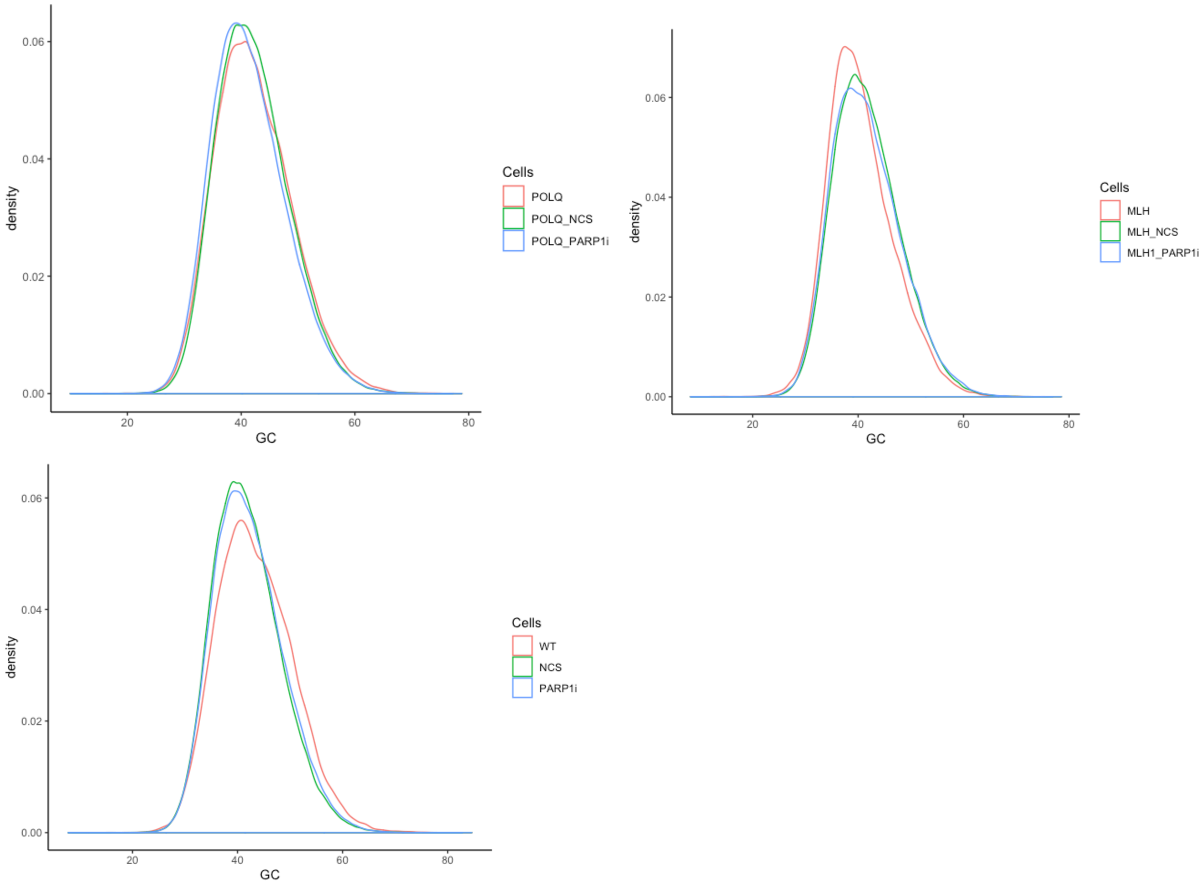

(**H**) % of microDNA that had microhomology of >1bp at the junction was comparable in the libraries prepared from WT and mutant U2OS cells and following treatment of select cells with various chemicals.

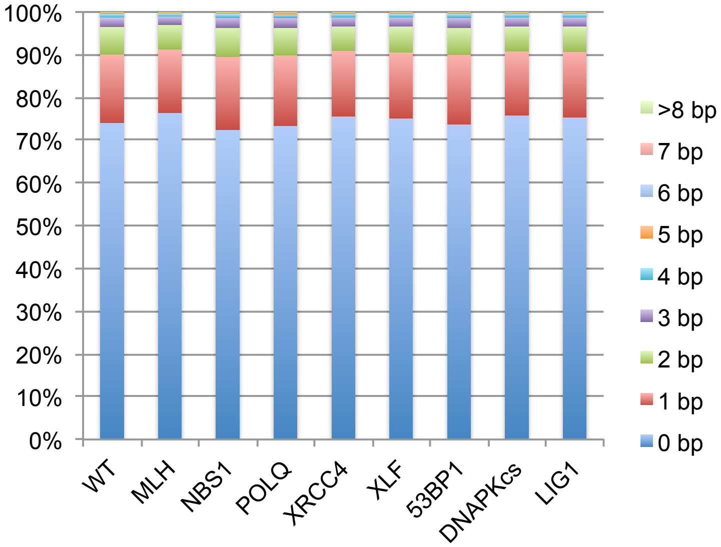

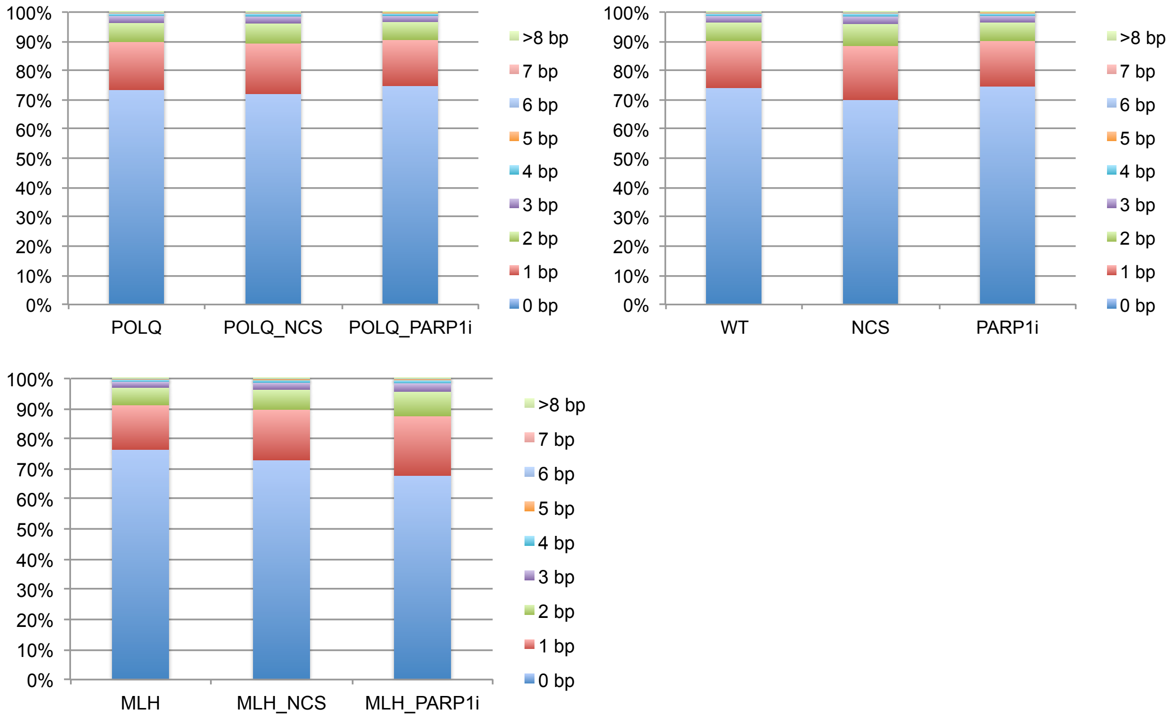

(**I**) Some abundant microDNA after NCS treatment in U2OS cells are derived from exons of genes relevant for cancer, so they could produce short regulatory RNAs that dysregulate these genes.

**Highly abundant microDNA carrying exon or promoter sequences**								
Abundance	Chromosome coordinates			Gene from which microDNA arose			Cancer type	PMID
1386	chr9	96490426	96491179	HABP4	Exon	Tumor Suppressor	Colorectal Cancer	33245729
345	chr5	141056511	141057242	PCDHB1	Exon	Circulating eccDNA	Prostate cancer	22619380
336	chr10	84172015	84172602	CERNA2	Exon/Promoter	Oncogene	Cervical Cancer	30793356
282	chr7	23199959	23200555	NUP42	Exon	Nuclear export	Normal tissue	28869701
203	chr7	99534321	99534585	ZKSCAN5	Exon	BRAF-fusion	Melanoma	23890088
194	chr15	68632317	68633467	CORO2B	Exon	Cytoskeleton	Normal tissue	29162887
178	chr2	101039952	101040937	TBC1D8	Exon	Oncogene	Ovarian cancer	30809301
156	chr1	37861131	37861440	INPP5B	Exon	Phosphatase	Normal tissue	23805271
135	chr6	131894095	131896292	ENPP1	Exon/Promoter	Pyrophosphatase	Many cancers	31752288
104	chr4	56805903	56806941	RN7SL357P	Exon/Promoter	Unknown		

(**J**) MicroDNA arise specifically from hotspot regions. The normalized abundance of the Q-PCR product (relative to mitochondrial DNA) obtained by outward facing primers interrogating different sites at a genomic locus. The hot-spot region has a much higher abundance of signal than the neighboring sites suggesting that a given set of primers is not missing circles emerging from adjoining regions with different junctions.

microDNA candidate 1

KIF26B

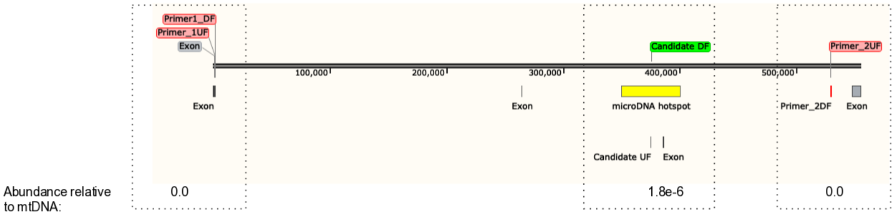

microDNA candidate 4

TJAP1

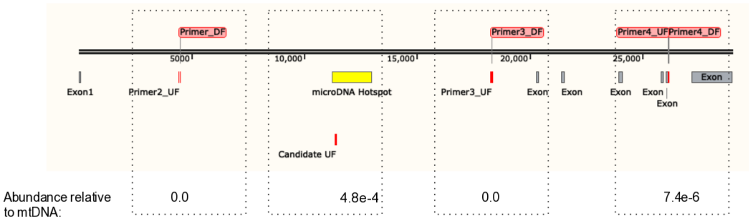

Supplemental Figure 2:
(A) Cutting efficiency of CRISPR/Cas9 system at Chr22 assessed by mismatch created by repair of cut. T7 endonuclease digestion of PCR amplicon after de- and re-naturation will cut mismatched duplexes. Amplicons were from chr22:18623440-18624398 after transfection of plasmid carrying CAS9/sgRNA targeting either chr22: 18624104 or chr12:117100086.
(B) ~60% of DNA amplicons were cut by T7 endonuclease, indicating a successful cut by CRISPR/Cas9 followed by erroneous repair by NHEJ.

A

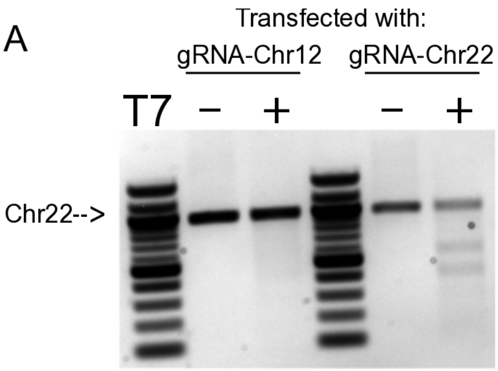

B

Efficiency of Chr22 cut

	Area
Uncut	56331
Cut band 1	43466
Cut band 2	37274
Percent Cleaved	58.90

Supplemental Figure 3:

(A-D) The efficiency of small molecule inhibitors utilized in study. Treatment of 293T cells decreased cell viability as expected from functional inhibitors. (A) Cisplatin (CIS) and NSC16168 (inhibitor of ERCC1-XPF), (B) D-103 (Rad52 inhibitor) or B02 (RAD51 inhibitor), (C) cisplatin and B02, (D) Mx (APE1 inhibitor in the BER pathway) with CrVI (hexavalent chromium).

(E) Mirin significantly reduces cell survival during 48 hour treatment. This indicates that treatment with Mirin for 48 hours is not useful for microDNA measurement because of the death which occurs in ~90% of cells.

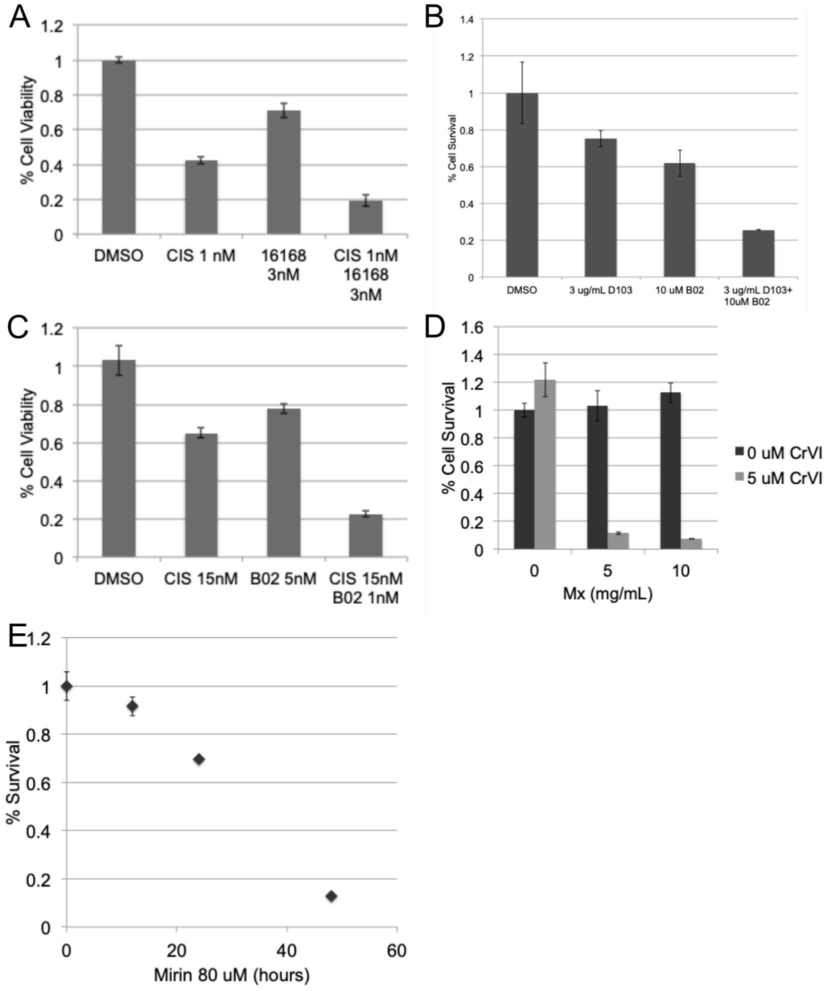

Supplemental Figure 4: Propidium Iodide FACS profiles to show cell-cycle profiles. (**A**) To ensure that cells were progressing through S-phase as predicted, we analyzed the cells at several points after release from hydroxyurea (HU) block: 0, 1.5, 3, 4.5, 6, 7.5, and 9 hours. We also confirmed that cells were stalling in M phase after the addition of nocodazole. (**B** and **C**) Cell cycle distribution of U2OS cell lines with mutations in indicated genes. (**D**) MicroDNA levels through S-phase after thymidine block and release. (**E**) FACS profile of cells after release from thymidine. (**F**) Mitochondrial DNA levels (used as normalization factor) through the cell cycle.

(**A**) FACS profile of cells during HU block and release (Unsynchronized, HU treatment (24 hours), Release (1.5, 3, 4.5, 6, 7.5, 9 hours).

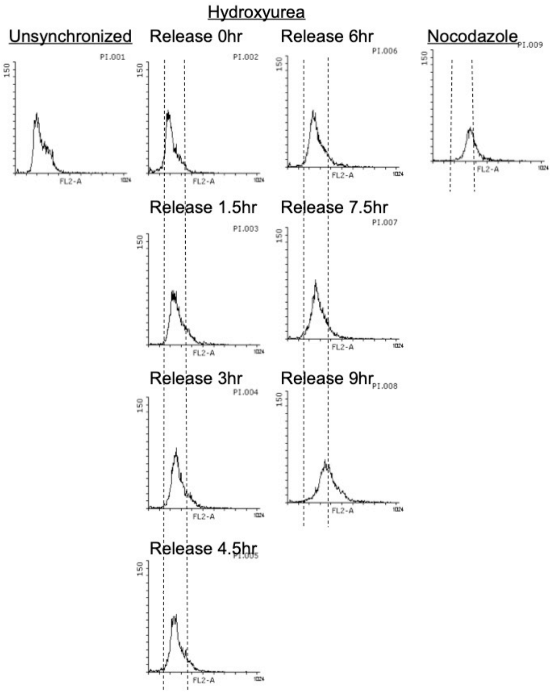

(**B**) Distribution of U2OS knock-out cell lines in the cell cycle phases.

U2OS KO	G1	S	G2
WT	27.6	49.5	22.9
53BP1	33.1	45.1	18.5
DNA-PKcs	36.2	45.6	14.4
LIG4	22.0	49.8	23.0
XLF	49.3	34.7	11.9
XRCC4	30.9	44.0	21.7
NBS1	28.9	43.4	27.8
MLH1	40.2	41.3	18.5
XRCC4	39.8	32.1	28.1
POLQ	33.2	43.0	23.9

(**C**) Distribution of U2OS knock-out cell lines in the cell cycle phases.

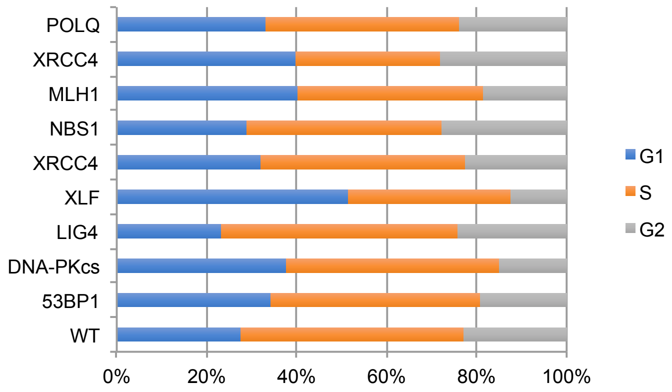

(**D**) MicroDNA levels as cells progress through S phase after thymidine block and release: Unsynchronized, Thymidine block for 24 hours without any release (0 hrs) and following release (3, 6 ,9 hours).

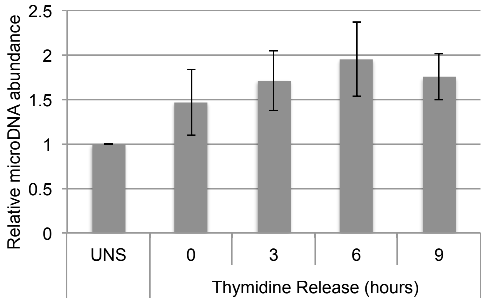

(**E**) Cell cycle profiles by propidium iodide FACS to show unsynchronized cells (UNS), G1 accumulation after Thymidine block (Thy) and progression through S phase upon release for 3, 6 and 9 hr (REL3, REL6, REL9)

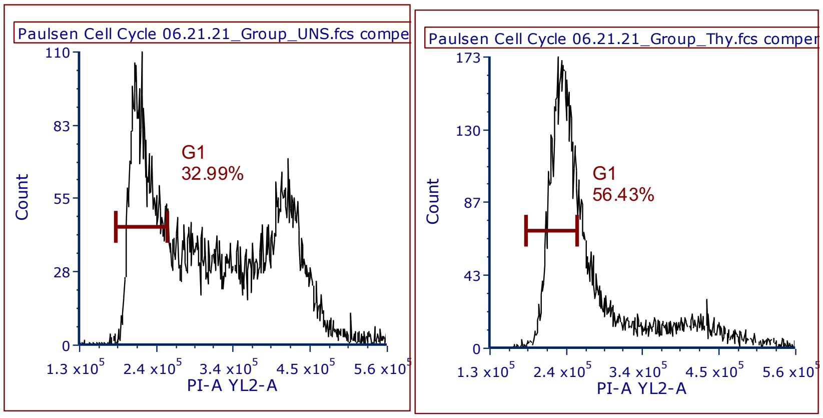

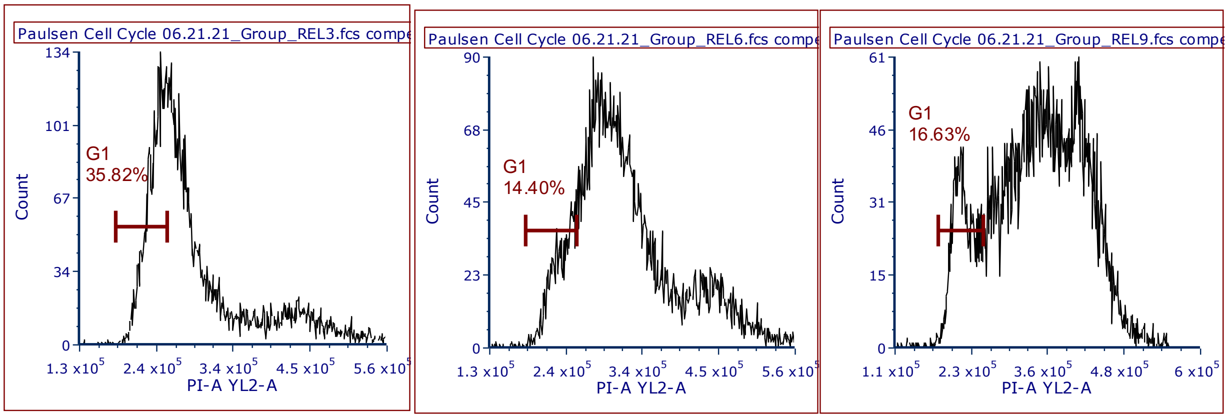

(**F**) Levels of mitochondrial DNA through the cell cycle are not highly altered relative to genomic DNA

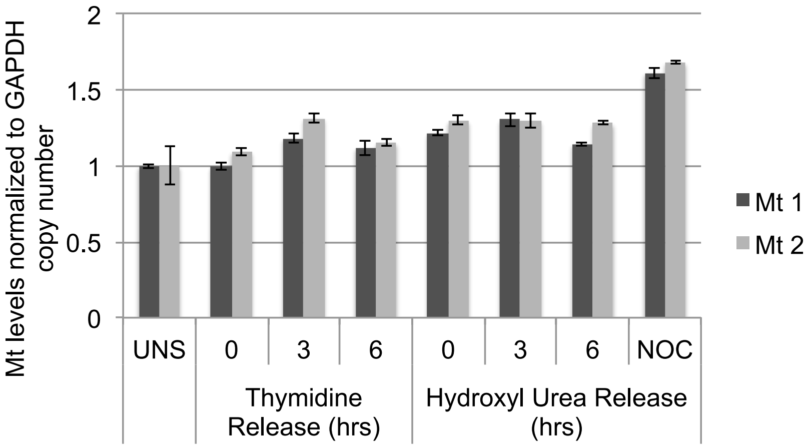

Supplemental Figure 5: Confirmation of U2OS cell lines
(A) Two single guide-RNAs (sgRNAs) targeting the different genes were cloned into the pX330 vector (Addgene #42230) containing a human codon-optimized SpCas9 endonuclease at two proximal (within 300 bp) sites in early exons of each gene. DH5 bacteria (Invitrogen) was used to amplify the vectors; the vectors were then purified using the QIAprep Spin Miniprep Kit (Qiagen) according to the manufacturer's instructions and tested using U6-specific primers by Sanger sequencing (Eurofins Scientific).

The Cas9/sgRNA plasmids, as well as the pMSCV vector containing the puromycin resistance gene (Clontech), were transiently transfected into human osteosarcoma U2OS cells using Lipofectamine 2000 according to the manufacturer's instructions (Invitrogen). Puromycin (2 g ml-1) was added to the cells 24 hours after transfection for a 48-hour incubation period, after which they were seeded at a low density.

Each clone's samples were lysed overnight at 55°C in lysis buffer supplemented with 20 g proteinase K (100 mM NaCl, 10 mM Tris-HCl pH 8, 25 mM EDTA, 0.5 percent SDS). Genomic DNA was extracted using phenol/chloroform/isoamyl alcohol and then precipitated with ethanol. PCR amplification of the targeted locus with gene-specific primers was accompanied by Sanger sequencing for genotyping. Immunoblotting was used to confirm the deletion of the different genes in the knockout U2OS cells.

Sg-RNAs (sense strand) used to create knock-out cell lines:
PRKDC: sg-DNA-PKcs-1: 5’-GAGCCGGTGTGCGTTGCTCC-3’, DNA-PKcs-2: 5’-GCCGGTCATCAACTGATCCG-3’; TP53BP1: sg-53BP1-1: 5’-GACGCACAAAGAAAATCCTG-3’, sg-53BP1-2: 5’-GAACGAGGAGACGGTAATAG-3’; LIG1: sg-LIG1-1: 5’-AGAGTGACTCTCCGGTGAAG-3’, sg-LIG1-2: 5’-TTAGCCCTGCTAAAGGCCAG-3’; POLQ: sg-POLQ-1: 5’-TGAATCTTCTGCGTCGGAGT-3’, sg-POLQ-2: 5’-GATTCGTTCTCGGGAAGCGG-3’; NBN: sg-NBS1-1: 5’-GCGTTGAGTACGTTGTTGGA-3’, sg-NBS1-2: 5’-TAACTTTTCTGTAACCAACC-3’; MLH1: sg-MLH1-1: 5’-TGATAGCATTAGCTGGCCGC-3’; sg-MLH1-2: 5’-CCCTGCCACGAACGACATTT-3’; MSH2: sg-MSH2-1: 5’-GAAGCGCACGAAGCCGACCT-3’; sg-MSH2-2: 5’-GGCGACTTCTATACGGCGCA-3’.
For immunoblotting the following antibodies were used:
Anti-DNA-PKcs (Abcam; # ab44815); anti-53BP1 (Novus Biologicals; # N100-304); Anti-POLQ (Abnova; # H00010721-M09); anti-LIG1 (Proteintech; # 18051-1-AP); Anti-Tubulin (Santa Cruz; # sc-53646); anti-NBS1 (abcam; # ab32074); anti-MSH2 (BD Biosciences; # 556349); and anti-MLH1 (BD Biosciences; # 554073).

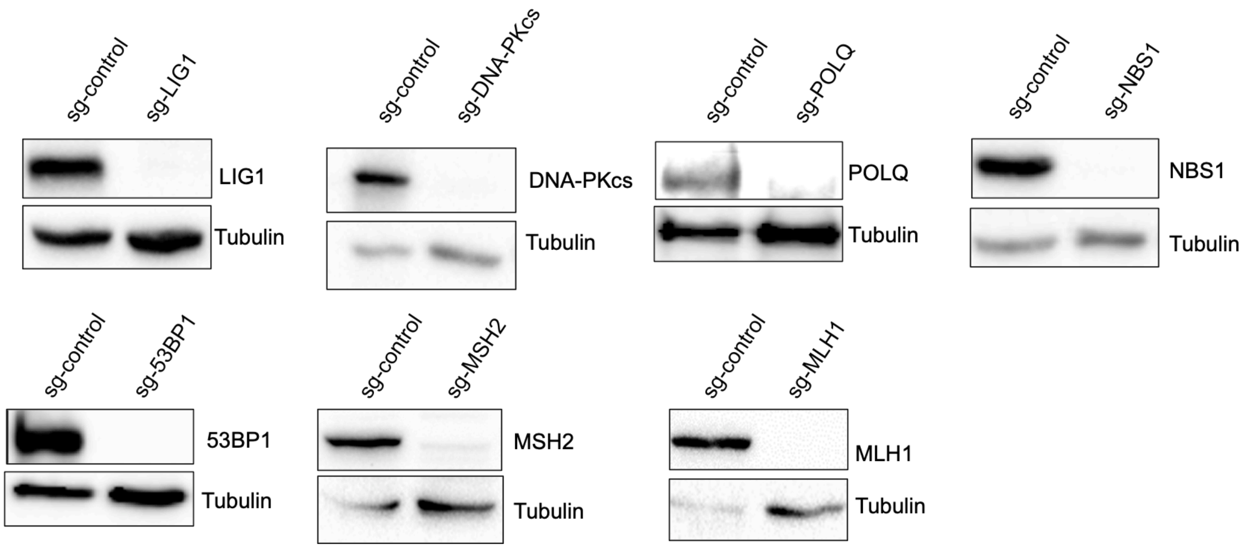

(B) Gamma H2AX western to show that changes in DNA damage response in the mutant cell lines do not explain the observed differences in microDNA levels.

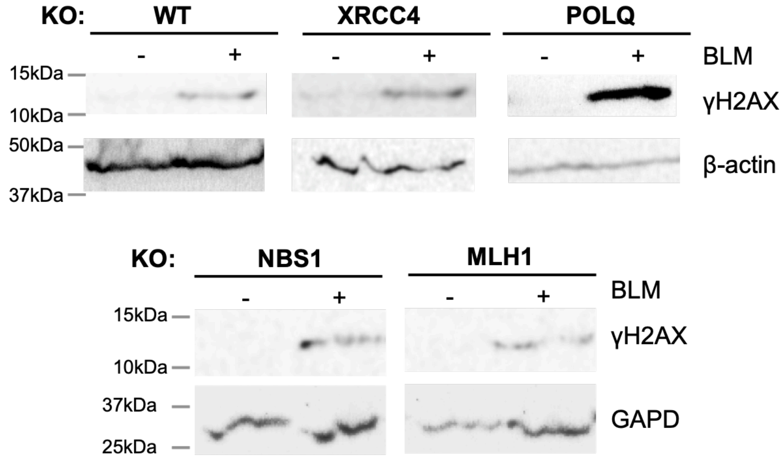

Supplemental Table 1: Chromosomal loci interrogated by inverse PCR to measure endogenous microDNA. These sites were selected because they produced highly abundant microDNA in human or chicken cells as detected in microDNA libraries prepared by rolling circle amplification and high throughput sequencing from past research.(14) The abundance was listed as the number of times the junction sequence was sequenced, thus giving an estimate of the percentage of the specific microDNA within the entire population of microDNA. Sequences of outward facing primers, size of inverse PCR amplicon and sequence of the amplicon with the two parts that are joined to form the circle are given. The two parts are indicated by bold and regular font; the junction is at the shift between bold and regular font. UF: Upstream facing, DF: Downstream facing.

A) Human microDNA. **B)** Chicken microDNA. Some primers amplified more than one microDNA, therefore some primers have more than one sequenced amplicon.

(**A**)

Human OF Primer and Amplicon Sequences				
**#**	**UF Primer sequence**	**DF Primer Sequence**	**Size of Amplicon (bp)**	**Sequence of OF primer PCR amplicon**
1	TACTACCATTGCTAGATTGC	GAGATAGCCAAGCTCCCATG	567	**GAGATAGCCAAGCTCCCATGTTTATTGCAGCACTATTCACAATAGTCAAGAGTTGGAAGCAATCTAAGCATCCATCAATAGACAAATGAATAAAGAAAAGGTGGTACATATACAGAATGGAGTACTCTTCAGCCATAAAGAAGAATGAGATCCTGCCATTTGCAACAACATGGATGGAACTGGAGGTCATTATGTTAAGTGAAATTAGCGAGGCCCAGAAAAACAGACTTCACATGTTTTTATGTGTGCTGCCCAGATGACACAATTCAGGAAAATCTTTCAGCTCCTACCAGAGAGTAAAGCTCTTCAGCTGTAGTGG**CTCAACATCATTCATTGATCATCATAGAAATGCAAATCAAAACTACAATGAGATTATCATCTCACCACAGTTAAAATGGCTTTTATCCAAAAATCAGGCAATAACAAATGCTGGTGAGGATGTGGAGAAAAGGGAACCCTCATACACTGTGGATGGGAATGTAAATTAGTACAACCACTGTAGAGAACAGTTTGGAGCTTCCTCAAAAAACTAACAATTGAACTACCACGCAATCTGCAATGGTAGTA
2	GGCCCTCTGGTGGACATCTCATTAA	CTGGTGGGCGCTATGGAATCTT	251	**CTGGTGGGCGCTATGGAATCTTGTCCCCTGGAGAGGCACACAGCCAGGGCAGAACATCAAGGTCAAGGCTCTCCTGAAGGCTCTGCAGTGCTTAGTGACACCACCATCAATGACCGTCAGGTATCAGGTCTGTTCTGAACGTTAGTCT**TCTCCATCCAGTTGGGGTGACTGGGGCCGATGCTGTTGCCTGTGTAATGTGATTCCTCCTCCTTAAAATAAGGATAAGTTAATGAGATGTCCACCAGAGGGCC
3	GCCTCCCAAGTAGGTGGGAT	GAGAATTGCTTGAACCCTGGAG	281	**GCCTCCCAAGTAGGTGGGAGCACACCCCACCATGCTCCAC**TAATTTTTGTATTTTTAGTAGAGACGGGGTTTCACCATGTTAGCCAGGATGGTCTCAATCTCCTGACCTCGTGATCTGCCCACCTTGGCCTCCCAAAGTGCTGGGATTACAGGTGTGAGCCACCACGCCTGGCTTTTTTTTTTTTTTTTGAGACAGAGTCTTGCTCTGTTGCCCAGGCTGGAGGGCAGTGGCACGATCTCTGGTCACTGCAACCTCCACCTCCAGGGTTCAAGCAATTCTC
4	ACCACCCTGGCCACAGAGGT	GCCCTGAGTCATCAGAGAGC	270	**ACCACCCTGGCCACAGAGGTGGGCCTGGCCTTTTCCCAAGCCTAGGATCAAGATTCACGCAGACAGGAAACACTTCCAATAATTGCCTACACACTGGGCTCCCCATGTTGCTCTGACTACAGCCCAAGAGCTCAAATAGGCCAAGGCCTGACTATGAAGA**TGATCCTAACTTCTTATCCTAGGGTGAGAGTAACAGGGACACTATCCAACCTTCCCTTCTTGATAAAGGGAGGAAATGATCTGATCTAGGAGAAAAGTCCCTTCAGTTCA
5	GATCACGAGGTCAAGAGATGGAG	CCTCTGAAAGTGCTGGGATTACA	122	**GATCACGAGGTCAAGAGATGGAGACCATCCTGGCTAACACGGTGAAACCCCATCTCTACTAAAAATACAAAAAA**TTAGCCGGGCGTGGTGGCGAGCGTCTGTAATCCCAGCACTTTCAGAGG
6	CATCGTTAACTGTCAGCGAC	TTTGAAGCAGACCAGAAGAG	919	**CCTCACTTAAACCTATCCCCTCCCACTACTGCCACCCCTGCAAAAGCTGAAAACTAAGCTGAGACTGACAATCTCAATCACAAATCGCATGCACAAGCAGAGGCGGCAAAGAGCCAAGCACCCACGGAGACTTGGTTAGTGAAGGTCTCCTCATTGACCGATGGTGGGGAGAAAAAGTGGAGAATATTTTCTGTCCTCTTCTGGTCTGCTTCAAA**CATCGTTAACTGTCAGCGACTCTGCCAGTGCAAAGGAAACATCTGTATCAGCCAAACCTGTTGAAGATTAAACAGTTAACTGAGTCTAAACACATATGCAAGTGAGAATACCAATGGCCCTAAAACAAAACCTGTGCATCGAAAACGCTTGATTGATGTTTCTATATCTTAGCATTCACTGCAAGCGCTGGGGTCATTTGAGGGTAGTTTAAATCTGTGGCTGCATTTCAGAGATGATTTTTAGTTCTTTAGATTGTTGATGTCAAAGATCAGTGTGGGAGGCAACTAGAATCAGTGAGCCTTGTCCTTGTCTTGAACTTTCTTCACCTTTCTCCATGGAGAGGAAACAATGAATTTTCTCAGCAGGGACTGTATTTTGAAATCTTCCCTGGAAATAATCACTAAGGAAAAAAATGTTTCTGGTAGGATGTTTCATGAATCACAAGTAAGTTTTTTTTAAAAAAATCAAAATCTTTTACTTCTACAAAAATTAGAACAGGTTGTTCCTCTCAGTGAAGTTTCTGAATTTATGTTTGTTGGAAAGGAAGGTTGAAAAATTCTGAAGAGAAGTTATATGCATATCCAGGACAATTTGAATAGGTGGAATGGTAGCCAGCCCAGTGAATCTCCGGAACATGGTCTGTGTGGGCTTAGCCATGATGTATGCTAGGGAATGGGTCAGGGAGGGATAGGTTTAAGTGAGG
7	CCCAACTTCTGGTGGCCTGTT	TGCATAGAAGGAAGTGAGT	134	**GCCCGGTACCTAACAGGCCACCAGAAGTTGGG**GGCCTGTGAGGAACCGGGCTGCATAGAAGGAAGTGAGTGGCAGGCAAGCTTCATCTGTATTTACAGCTGCTTCCCAATCGCATACATTACTGCCTGAGCTCC
8	GAGAATTGCTTGAACCCTGG	GCTCCCAAGTAAGGTGGGAC	988	**GAGAATTGCTTGAACCCTGGAGGCGGAGGTTGTAGGGAGCCAAGATCGTGCCATTGCACTCCAGCTTGGGCAATAAGAGCGAAACTCCGTCTCAAAAAAAGAAAAAAGGAAATATACATATAGCCGGGCGTGGTGGCTCACGTCTGTAATCCCACCTACTTGGGAGGCCGAGGCGGGTGGATCACCTGAGCTCCGGAGTTCGAGACAAGCCTGGGCAACATGGCGAAATCCTGCCTCTACAAAAAATTAGCTGGGCCTGGTGGCGCATGCCTATAATCCCACCTACTTGGGAGGCTGAGGCAGGAGAATTGCTTGAACCCTGGAGGCAGAGGCTGCAGTGTGCCGAGAACGCGC**CATCGCGATTGTAAAACCTAAGATTGGCACTTGAGGTATTTCTCAGACCCTGCACTGCTGATGCACTCTGATGCACAAACTGGTGCCACCCAGGCCAGTAATCTGGCTCAACCAGTTCTGCGATCCTACCCAGGAACAGAAGACAGCAAGAATCCACTTCAGGCGGATGATTCACGCCTGTAATCCCAACACTTTGGGAGGCCGAGGTGGGCAGATCATTTGAGGTTAGGAGTTCGACACCAGCCTGGCCAACATGGTGAAACCTCATCTTTACTAAAAATACAAAAATTAGCCGGGCATGGTGGCATGTGCCTGTAGTCCCAGCTACTTGGGAGGCTGAGGCAGGAGAATTGCTTGAACCCTGGAGGCGGAGGCTGCAGTGAGCCGAGATCGCACCACTGCATTCCAGCCTAGGTGACAATAGCAAAACTCAGTCTCAAGAAAACAAAAATATTTTTAGAGATGGGGGGCTTTCTATATTGCCTAGACTGGCCTTAAACTCCTGGCCTCAAGTCACCTGAGTCAGGAGTTCAAGACCAGCCTGGCCAACATGGTGAAACCCCGTCTCTACAAAAAATACAAGAAAATTAACCAGCGTGTGGCACATGCCTGTAGTCCCACCTTACTTGGGAGC
9	AACCTCCACCTCCCGG	GACCCTGTCTCTAAAAAACAAAAAA	335	AGCCTCCACCTCCCGGATTTAAGCAATTCTGCCTCAGCCTCCCGAACAGCTGGGATTACAGGCATGAGCCACCACACCTGGCTAATTTTTGTATTTTTAGTAGAGGCGGGGTTTCGCCATGTTGGCCAGGCTGATCTCGAACTCCTGACCTCAGATCAACAGATTCAACAAGACCCAGCAAGCTTTTTGTAGAAATTAACAAGAAAGGTTATGAGTGGGTAACTGAAGCTGGGTAACGTGTACCTGAGAGTTGATTATACTACTCTCTTCACTTTTGTTTATGTTTGAAATCATCCATAATAAAGTGTTGTTTTTTGTTTTTTAGAGACAGGGTC
10	CAGCTCACAGCAGTGTCCCT	GCTCACAGCAACCTCCACCT	401	CAGCTCACAGCAGTGTCCCTCGCCAGGAGTTGCCAACCTATAAAGGAAGCTGCCATGACCTAGGCTGCGACCTCTCCCTAGAGGCAGCCAGCATCCCACAACTGGGTGACTCAGGAGTGCAAAGACTCAGCCCCTGTCCTCAACCATGCATAAGTCTGAAAGGCCATTCCAGGCTGGGCACAGTGGCTCACACCTGTAATCCCAGCACTTTGGGAGGCTGAGGCGGGAGGATCACCTGAGGTCAGGAGTTCGAGACCAGCCTGGCCAATGTGGTGAAACCCCATCTCTACTAAAAGTACAAAAAGTAGCTGGGCGTGGTGGTGCACGCCTGTACTCCCAGCTACTCCGGAGGCTGAGGCAGAAGAATCGCTTGAACCCAGGAGGTGGAGGTTGCTGTGAGC
11	GGCACCATCTAGTCAGCTGC	ACACTGGCAGCTGACTAGAT	581	GGCACCATCTAGTCAGCTGCCAGTGTGGTTAGAATAAAAGCAGGCCGCAGAACGTGGAAGGACTTGACTTGCTGAGTCTTCCGGCCTTCATCTTTCTCCCGTGCTGGATGCTTCCTGCCCTTGAATATCAGACTCCAAGTTTTTCGGCTTTTGGACTCTTGGACTTACGCCAGTGGTTTGCCAGGGGCTCTTGGGCCTTAGGCCACAGACCGAAGGCTTCACTATAATGCAGCCTCCCATAACCAAAAATCACAGATCGGGTGTCTGGATGTGTGTGATAAGTGAGGTTCCCTAAGGGTCTAAACATACAGACTTTCAACTAGGGTCCCTATGTTAAGCCCCATGCTTAAGCTTACCCAGCTTTTGCTGCATCTGATGCCTCCAATTCCCGACTATGTTAGTTGGGGCTGTGTGATGGTTAATACCGAGTGTCAACTTGATTGGATTGAAGGATGCAAAGTATTGTTCCTGGGTGTGTCTGTGAGGGTGTTGCCAAAAGAGATTAATATTTGATTCAGTGGACTGGGAGAGGCAGACCCACCCTCAATCTGGGTGGGCACCATCTAGTCAGCTGCCAGTGT

	Primer F	Primer R
Mt	GATCAGGACATCCCGATGGTG	AGGCGCTTTGTGAAGTAGGC

(**B**)

DT40 OF Primers and Amplicon Sequences

#	UF Primer sequence	DF Primer Sequence	Coordinates of eccDNA by NGS			Abundance by NGS	Size of circle from NGS (bp)	Coordinates of sequence in genome	Size of Amplicon (bp)	Sequence of OF primer PCR amplicon
1	ATGCAAACCTGCCTTCGTTC	GCAGTGGTATGGGACCTGTC	chr11	2300736	2300935	19280.6	199	Chr11: 2300736 - 2300969	200	**GCAGTGGTATGGGACCTGTCACAGGAACCATATGGTCGTCTGCTTCTTCTGCAAAGCAGCCGTCACAAGAGAGGAGGATGCTGCA**GGGCTGCATGAATAAGCAGTGAGGAGCAAACCCACAAGGAAATTCAGGCAGTGCTTGGTTTTGCATAACAGTGCTATTTTGTCTGAGTTGTGGTTTACTGTTATGTTGTACAAGAAGAGACTATCGGATGCAAAGCCTCAACTCTGTAACTGGGAGGAGAGAGGGACAGCCATTACATCCATCAGTGAAACTGGAGGAGATGATGAACGAAGGCAGGTTTGCAT
1	ATGCAAACCTGCCTTCGTTC	GCAGTGGTATGGGACCTGTC	chr11	2300736	2300935	19280.6	199	Chr11: 2300736 - 2300969	330	**GCAGTGGTATGGGACCTGTCACAGGAACCATATGGTCGTCTGCTTCTTCTGCAAAGCAGCCGTCACAAGAGAGGAGGATGCTGCATATTGCTGCTGCCTCTGGAACGACGATATTTAGGT**CATCCATCAGTGAAACTGGAGGAGATGATGAACGAAGGCAGGTTTGCAT
2	TAAGCCTCAATGTGTGTTCAGCT	GAGGCAATTCTGAAAGAAGCCG	chr4	1460467	1460815	8535	348	Chr4: 1460467 - 1462710	174	**AAGCCTCAATGTGTGTTCAGCTGGAATGCTGTCTAGCTATACATCCCTTATTAATGCCATATTAACAGACACAGCTGTTCAATATGCAGTATGCAAACACACATGGATGTAAACAATCAGAGCAGAGGAGAGCTTTTGTTTCCTCGGCTTCTTTCAGAATTGCCTCCTCACTGTAAGCCTCAATGTGTGTTCAGCT**GGAATGCTGTCTAGCTATACATCCCTTATTAATGCCATATTAACAGACACAGCTGTTCAATATGCAGTATGCAAACACACATGGATGTAAACAATCAGAGCAGAGGAGAGCTTTTGTTTCCTCGGCTTCTTTCAGAATTGCCTC
2	TAAGCCTCAATGTGTGTTCAGCT	GAGGCAATTCTGAAAGAAGCCG	chr4	1460467	1460815	8535	348	Chr4: 1460467 - 1462710	180	**TAAGCCTCAATGTGTGTTCAGCTGGAATGCTGTCTAGCTATACATCCCTTATTAATGCCATATTAACAGACACAGCTGTTCAATATGCAGTATGCAAACACACATGGATGTAAACAATCAGAGCAGAGGAGAGCTTTTGTTTCCTCGGCTTCTTTCAGAATTGCCTCCTCACTGTAAGCCTCAATGTGTGTTCAGCT**GGAATGCTGTCTAGCTATACATCCCTTATTAATGCCATATTAACAGACACAGCTGTTCAATATGCAGTATGCAAACACACATGGATGTAAACAATCAGAGCAGAGGAGAGCTTTTGTTTCCTCGGCTTCTTTCAGAATTGCCTC
3	AGGAGCATCAACACTGATGCA	CCCCTAGATGGTCTGGACCT	chr3	25576742	25577047	5161.2	305	Chr3: 25576742 - 25577047	205	**CGTCTTGCTATAGCCCCCATCACCAACACACCTCTCACATTCCTCAAAATGCACCTTGAGACCAGAAACGAGCCCCCCTTGTTACCACGAAGCCATAACAAGCCCACAGCAACCACGTGCTGTCAGAGGCAGTGCCCAGGAACCCCAACCATGTCTCCCCAGCCTT**TGTCACTTGAAAGCTCACTGCATCAGTGTTGATGCTCCT
3	AGGAGCATCAACACTGATGCA	CCCCTAGATGGTCTGGACCT	chr3	25576742	25577047	5161.2	305	Chr3: 25576742 - 25577047	170	**CCCCTAGATGGTCTGGACCTTTATGCATAGACCTGATGCAGTGCTTTTTCAAATGCCTGAAATTGATAAAAGGATTCTGGTGGATTTTGGCCCCAAGCAGGCAGATGCCTCCTGTATACACAGTGTGCTGTACAGCTTCTACTGCATCTC**ACTGCATCAGTGTTGATGCTCCT
4	CTGCACTGTGATCAGCAGCT	GGAGGACAGATGGCTGCAC	chr4	82442526	82442718	5096.5	192	Chr4: 82442326 - 82443624	177	**GGAGGACAGATGGCTGCACCGCAGTCACTGCCAGCTGACACCTGCACAGGT**TGAGCTCTCAGAGCTCCCCGTCACCATTTCTTGCCTTCTGATCAGCAGTGTGCCTGTGAGTGAGAACTCACACGCAGCCCTTTTCAGGGCCCACGGGAGATGGAGCTGCTGATCACAGTGCAG
4	CTGCACTGTGATCAGCAGCT	GGAGGACAGATGGCTGCAC	chr4	82442526	82442718	5096.5	192	Chr4: 82442326 - 82443624	179	**GGAGGACAGATGGCTGCACCGCAGTCACTGCCAGCTGACACCTGCACAGGTTGAGCT**CTCAGAGCTCCCCGTCACCATTTCTTGCCTTCTGATCAGCAGTGTGCCTGTGAGTGAGAACTCACACGCAGCCCTTTTCAGGGCCCACGGGAGATGGAGCTGCTGATCACAGTGCAGCTCTC
4	CTGCACTGTGATCAGCAGCT	GGAGGACAGATGGCTGCAC	chr4	82442526	82442718	5096.5	192	Chr4: 82442326 - 82443624	303	**TGGCTGCACCCTCCCTGTCCGACTCATCCTCTTCAGACAGCCCCGAGTCAGC**TTCGGTGGGAGCTGGATCTGGTGGGGCAGCCTTGACGGGGGGCTCAGGCTCAGCTGAGTCCAAGCTGGGCTCCACTATGTGGAGCTCGGTGCTGTCCAGCTCTGCCTCTCCTTCCTCCTCCTCCTCTTCCGAGCTCTCAGCCTCTTGCTGAGCCACAGGACCCCCCGGGCATGGAGCCCCCTCTGCTCCCCAGCT
5	TTAGTTTCTGCTCGGCAGCA	TCAAACTCCGAGCACCAGTC	chr2	69137247	69137609	1057.7	362	Chr2: 69137047-69137904	360	**TCAAACTCCGAGCACCAGTCGTCTCGCTGTCAGGCAGTGCATGCTGGCTGCCTGCCTTATGGTGTGTGG**AGCACCAAGGCAGACAGTAGTTCAGAGCACTTTGCTGTTGTAAAATAAGGTTTCAAGCTCAGAGACACTGGTTTGCGCTCTCAAAGATGCAGATTCTGATCATCATCACATTTCATCTCGGTGATCTGTATCACTGAGTCCCAACAGTATGTAAGTTTGCATCAGATTCTCCCCTAAGTTCACTTCTTGCTTAGTTCACTGTTATGCTGCAGGTCTTTTTTTTTTAACCCTTCCATTGAGGGAGGTAAATGTAAAATGCTCAAGTGACTGCTGCCGAGCAGAAACTAA
6	ACTTCATCCAGTCCAGTGACAG	GAGGCTTCCACAACCTTTCAGT	chr8	13132838	13132985	1014.2	147	Chr8: 13132654 - 13133032	335	**ACTTCATCCAGTCCAGTGACAGCAGTACATCAGCAGATCCATACTGGCGTATCCTGCTGGTT**CTTCACACCTGTAGGTTGCCAGTTTTAGTCTGATCAGCCTAAAATGTTTTTTTTCAGTTGCACATGTGCTTTAGGACAGGTGTTGTACTGAAAGGTTGTGGAAGCCTC
7	GGGACAGGCTGAGAGCAGAG	GGCTTGGGAAGCAGTCATGC	chr2	20383499	20383673	1014.2	174	Chr2: 20383321 - 20383744	199	**GGCTTGGGAAGCAGTCATGCACATTG*GCC*TACCTGGCATGGGATTTGAACATCTTCACCCACAGTGTAAGACAGTGTGCATTGTGATGTGTAACTCCCATTGTTTTTTCTGCTATTTGCAGTGTAACGTGTGGATTAGTGCCCAAGTCATCTCTGCTCTGCTCTCAGCCTGTCCCTAATGGCTTGGGAAGCAGTCATGCACATTG**GCCTCAGCTGCTCTGGTTCCTACCTGGCATGGGATTTGAACATCTTCACCCACAGTGTAAGACAGTGTGCATTGTGATGTGTAACTCCCATTGTTTTTTCTGCTATTTGCAGTGTAACGTGTGGATTAGTGCCCAAGTCATCTCTGCTCTGCTCTCAGCCTGTCCC
7	GGGACAGGCTGAGAGCAGAG	GGCTTGGGAAGCAGTCATGC	chr2	20383499	20383673	894.1	174	Chr2: 20383321 - 20383744	379	**GCTTGGGAAGCAGTCATGCACATTGGCCAATTCACAGGCTGAAGAAGTGCAGAGGGCAGTACTGGGCACTTAACATTGCATTCTGTTAGAGTAATTTTTATCAGCGCGCTGTAAGTACACAATTTGGCATAACTAGTGCCTGGCGCTGCCTGATCCCTCACTCAGCTGTCAGT**CCATCTCCATCTCTGACCAGACAGGTGGTTGGCTGCCTCAGCTGCTCTGGTTCCTACCTGGCATGGGATTTGAACATCTTCACCCACAGTGTAAGACAGTGTGCATTGTGATGTGTAACTCCCATTGTTTTTTCTGCTATTTGCAGTGTAACGTGTGGATTAGTGCCCAAGTCATCTCTGCTCTGCTCTCAGCCTGTCCCTAATGGCTTGGGAAGCAGTC
7	GGGACAGGCTGAGAGCAGAG	GGCTTGGGAAGCAGTCATGC	chr2	20383499	20383673	894.1	174	Chr2: 20383321 - 20383744	375	**GGCTTGGGAAGCAGTCATGCACATTGGCCAATTCACAGGCTGAAGAAGTGCAGAGGGCAGTACTGGGCACTTAACATTGCAT**TCTGTTAGAGTAATTTTTATCAGCGCGTTGTAAGTACACAATTTGGCATAACTAGTGCCTGGCGCCTCATGTCACAAACGCTCTCAGCCACAGCCATCTCCATCTCTGACCAGACAGGTGGTTGGCTGCCTCAGCTGCTCTGGTTCCTACCTGGCATGGGATTTGAACATCTTCACCCACAGTGTAAGACAGTGTGCATTGTGATGTGTAACTCCCATTGTTTTTTCTGCTATTTGCAGTGTAACGTGTGGATTAGTGCCCAAGTCATCTCTGCTCTGCTCTCAGCCTGTCCC
8	CACGACTGACAGGATTATCAGGC	AAGGATTTGTGCATGCTTCTGAC	chr1	30701201	30701303	894.1	102	Chr1: 30701201 - 30701303	190	**CACGACTGACAGGATTATCAGGCCCACAGTATCGCTCCTATCCCTTTTTCAGCAATGCCGCTTTCCAGGTTATTCCCTCACAGCGAATACCTGTTTT**ACAAAGGCTATATACCATACTGTAAACTGAGGCCTGTTCTCCCTTGTCAGAAGCATGCACAAATCCTTTTTATATGAAGGGGAGTTGGGTGCA
Mitochodrial DNA F:ACCCCCAACTCAAACACTCG R:GGTGGTTCCTAAGACCAACGG										

Supplemental Table 2: Abundance of microDNA normalized against mitochondrial DNA and normalized to control (WT or DMSO treated) 293T cells treated with **(A)** DSB-inducing agents and **(B)** CRISPR/Cas9 plasmids cutting at specific genomic site **(C)** DT40 knock-out cell lines. **(D)** U2OS knock-out cell lines and 293T cells treated with small molecule inhibitors. U2OS knock-out cell lines and 293T cells treated with small molecule inhibitors combined with: **(E)** DSB-inducing agents or **(F)** PARP1i. **(G)** U2OS cells treated with cell cycle stalling agents.

(**A**)

DNA damaging agents, Replication stalling, APE1i, DNAPKi											
		DMSO	UV	Cisplatin	MMS	X-ray	NCS	APH	APE1i (Mx)	DNAPKi	BLM
REP1	1	1	2.384	1.329	3.739	1.296	2.872	0.198	0.909	1.944	2.702
	2	1	2.023	2.252	1.986	4.464	1.532	0.749	2.467	1.670	2.386
	3	1	2.891	1.994	2.059	3.404	2.367	0.978	0.662	3.219	0.955
	4	1	2.134	8.185	1.396	1.659	2.094	0.362	0.568	1.428	0.986
	5	1	2.472	1.562	3.201	1.332	2.234	0.363	0.655	3.632	1.492
	6	1	2.045	1.375	3.475	1.919	1.098	0.195	0.804	1.841	1.022
	7	1	2.308	1.845	1.757	1.414	2.396	0.458	0.819	1.251	1.440
	8	1	2.652	1.558	1.719	4.257	2.580	0.199	2.351	1.710	1.518
REP2	1	1	2.595	1.111	3.989	2.840	2.387	0.171	1.289	3.424	1.995
	2	1	1.416	1.540	2.508	2.692	1.712	0.625	3.081	3.600	1.643
	3	1	1.381	3.852	2.099	2.221	4.026	0.836	0.289	2.717	1.492
	4	1	1.163	1.000	1.396	2.125	2.606	0.285	1.120	1.873	1.440
	5	1	2.472	2.697	5.260	2.507	2.725	0.287	1.520	2.306	2.671
	6	1	2.045	2.161	0.301	2.414	2.947	0.168	1.050	4.650	1.930
	7	1	1.610	1.013	5.029	3.348	2.709	0.374	1.212	5.982	1.542
	8	1	2.819	0.921	3.237	1.498	2.523	0.151	2.871	4.655	2.110
REP3	1	1	2.308	2.380	2.399	1.568	1.993	0.323	1.400	6.810	1.980
	2	1	2.526	5.461	1.660	1.729	2.076	0.296	1.262	1.717	1.376
	3	1	3.097	1.776	2.028	3.166	2.650	0.623	0.370	3.870	2.678
	4	1	2.606	2.659	2.833	2.064	2.608	1.691	0.215	4.918	2.084
	5	1	2.227	2.986	1.777	2.289	3.001	0.390	2.585	1.521	3.459
	6	1	3.093	2.527	2.431	1.663	1.315	0.327	1.092	4.755	4.577
	7	1	3.297	2.039	2.419	2.036	1.051	0.264	1.121	5.448	5.307
	8	1	2.063	2.562	2.205	1.701	1.045	0.229	1.576	4.293	3.247

(**B**)

MicroDNA arising at site of an induced DSB									
		150	400	800	1500	5000	10000	Chr12 + gRNA-Chr12	Chr12 + gRNA-Chr22
REP	1	1.507	2.367	2.688	0.898	0.848	0.824	1.572	1.010
	2	1.549	1.510	1.455	1.416	1.379	1.191	2.505	0.861
	3	1.468	3.798	2.215	0.990	1.015	1.063	1.904	1.227

(**C**)

MicroDNA in the DT40 Knock-out Cell Lines												
		WT	Ku70	LIG4	NBS1	FAN1	MSH3	FEN1	RAD54	BRCA1	BRCA2	CtIP
REP1	1	1	0.056	0.289	0.026	0.381	0.065	1.324	0.350	0.947	0.122	0.285
	2	1	0.179	1.012	0.053	0.825	0.180	0.501	0.826	1.887	0.513	1.720
	3	1	0.912	2.400	0.012	0.049	0.140	0.990	0.555	0.111	1.004	3.741
	4	1	0.320	1.045	2.653	0.080	0.233	0.258	0.348	0.759	0.069	0.532
	5	1	0.014	4.305	0.129	0.454	0.085	1.344	0.236	0.473	0.711	0.714
	6	1	0.163	2.379	0.063	0.386	0.041	1.111	0.177	1.279	0.215	2.377
	7	1	0.205	1.013	0.125	0.996	0.111	4.192	0.408	0.740	0.250	0.313
	8	1	1.867	2.400	1.005	0.269	1.577	0.069	0.591	0.101	0.177	0.070
REP2	1	1	0.162	0.816	0.014	0.032	0.009	4.562	0.760	0.763	0.256	0.124
	2	1	0.241	2.516	0.048	0.038	0.034	0.715	1.107	0.221	0.439	0.307
	3	1	0.209	2.313	0.036	0.004	0.001	8.464	0.555	0.411	0.924	0.437
	4	1	0.105	1.153	0.123	0.032	0.113	6.083	0.794	0.299	0.888	0.130
	5	1	0.001	4.286	0.014	0.020	0.028	0.352	0.414	1.198	0.348	0.336
	6	1	1.620	3.238	0.154	0.003	0.030	1.191	0.908	0.276	0.597	0.655
	7	1	0.774	2.412	0.207	0.013	0.031	1.173	1.057	0.632	0.117	0.172
	8	1	0.032	0.969	0.083	0.019	0.017	0.445	0.591	0.238	0.327	0.204
REP3	1	1	1.066	1.590	0.015	0.058	0.018	0.192	0.017	0.238	0.067	0.596
	2	1	0.373	1.641	0.051	0.569	0.136	0.715	0.036	0.684	0.539	0.655
	3	1	0.636	2.711	0.009	0.319	0.030	0.879	0.026	0.589	2.613	0.172
	4	1	0.437	0.836	0.057	0.084	0.039	0.192	0.090	0.121	0.144	0.059
	5	1	0.184	1.956	0.036	0.252	0.051	0.800	0.037	1.178	0.597	0.124
	6	1	1.015	1.127	0.009	0.174	0.004	0.350	0.003	1.142	1.000	0.307
	7	1	1.685	4.326	0.087	0.488	0.032	1.088	0.118	0.172	0.264	0.437
	8	1	1.107	1.059	0.029	0.209	0.029	0.391	0.045	0.042	0.165	0.130
REP4	1	1	0.106	1.155	0.014	0.305	0.217	1.324	0.025	1.725	0.122	0.151
	2	1	0.744	2.761	0.048	0.012	0.280	0.501	0.060	2.109	0.513	0.154
	3	1	0.196	4.400	0.036	0.266	0.103	0.990	0.047	2.529	1.004	0.322
	4	1	0.550	4.576	0.123	0.261	0.280	0.258	0.012	0.637	0.069	0.120
	5	1	0.007	2.298	0.014	0.057	0.231	1.344	0.025	1.505	0.711	0.996
	6	1	1.400	1.071	0.154	0.334	0.456	1.111	0.265	1.188	0.215	1.288
	7	1	0.073	8.653	0.207	0.857	0.123	4.192	0.198	3.426	0.250	4.304
	8	1	0.231	6.237	0.083	0.325	0.119	0.069	0.094	1.264	0.177	1.223
REP5	1	1	0.020	0.677	0.015	0.381	0.082	0.350	0.097	0.036	2.109	0.214
	2	1	0.282	1.404	0.051	0.825	0.136	0.882	0.059	0.129	2.529	0.266
	3	1	0.352	11.299	0.009	0.049	0.086	0.796	0.555	0.046	0.637	4.371
	4	1	0.106	0.739	0.057	0.080	0.096	0.352	0.361	0.078	1.505	0.339
	5	1	0.715	1.013	0.036	0.302	0.005	1.191	0.013	0.125	1.188	9.077
	6	1	0.036	2.067	0.009	0.454	0.104	1.173	0.093	0.053	3.426	0.927
	7	1	0.129	2.866	0.087	0.386	0.020	0.359	0.284	0.010	1.264	1.067
	8	1	0.162	1.984	0.029	0.020	0.048	0.445	0.068	0.037	0.036	0.300

(**D**)

Abundance of microDNA in U2OS knock-out cell lines, 293T small molecule inhibitors																
		WT	XRCC4	DNA-PKcs	53BP1	XLF	LIG4	NBS1	POLQ	MSH2	MLH1	LIG1	RAD51i (B02)	RAD52i (D103)	ERCC1/ XPFi (NSC16 168)	PARP1i (AZD 2461)
REP1	1	1	1.198	4.293	8.405	1.560	2.796	0.299	0.682	0.391	0.029	0.496	3.406	0.590	0.688	0.147
	2	1	5.944	3.659	1.908	1.987	0.607	0.406	0.051	0.031	1.690	0.403	0.160	2.262	1.130	0.934
	3	1	1.214	2.306	1.084	1.146	7.138	0.185	0.585	0.378	0.100	0.327	1.066	0.482	0.723	0.132
	4	1	1.306	3.219	7.194	2.809	3.524	0.202	0.536	0.183	0.018	0.188	1.257	0.356	10.735	0.269
	5	1	0.130	3.600	8.028	0.711	3.258	0.435	0.300	0.887	0.018	0.010	2.433	0.451	0.754	0.046
	6	1	7.213	3.870	0.371	0.535	2.611	0.515	0.155	0.138	0.005	0.308	2.081	0.495	0.475	0.144
	7	1	6.336	3.424	1.493	1.168	2.684	0.256	0.605	0.495	0.022	1.708	1.334	0.393	0.303	0.260
	8	1	1.463	3.632	2.753	0.509	3.181	0.290	0.569	0.323	0.069	1.205	1.385	0.910	0.904	0.288
REP2	1	1	1.724	1.587	1.663	0.921	4.380	0.237	0.576	0.415	0.693	5.023	0.487	1.228	1.251	0.363
	2	1	2.038	2.717	1.236	1.948	1.906	0.946	0.298	0.083	0.362	4.945	1.762	0.559	0.588	0.191
	3	1	2.492	4.918	8.405	1.214	6.032	0.162	0.133	0.076	0.188	1.460	1.275	0.960	0.525	0.240
	4	1	2.045	5.448	8.028	1.106	5.351	0.313	0.152	0.176	0.448	0.884	0.261	1.350	1.650	1.161
	5	1	1.591	4.755	0.970	1.306	3.618	0.168	0.173	0.428	0.624	0.003	0.343	1.069	1.289	0.729
	6	1	1.202	5.982	4.646	0.004	4.345	0.186	0.110	1.727	0.754	1.961	0.304	1.141	0.861	0.729
	7	1	1.417	4.650	0.455	2.186	2.399	0.227	0.252	0.343	0.561	0.152	0.428	1.420	1.049	0.270
	8	1	1.951	0.535	0.420	2.113	4.866	0.299	0.253	1.204	0.649	1.293	2.141	0.039	0.917	0.299
REP3	1	1	0.927	5.334	0.455	1.357	4.625	0.237	0.676	0.639	0.880	0.279	1.658	1.398	1.183	0.709
	2	1	0.715	1.525	1.525	0.987	5.542	0.248	0.289	0.500	0.065	0.219	1.448	0.955	0.387	0.863
	3	1	0.962	2.807	4.595	2.845	2.753	0.097	0.325	0.592	0.140	0.321	0.805	0.675	1.765	0.860
	4	1	1.069	1.199	4.065	0.944	4.671	0.230	0.480	0.951	0.057	0.565	1.409	0.919	1.322	0.843
	5	1	1.569	1.329	1.767	1.048	0.713	0.203	0.237	0.565	0.132	0.055	0.751	0.905	1.203	0.701
	6	1	1.750	4.441	0.420	1.001	7.587	0.214	1.095	0.298	0.086	0.717	1.868	1.742	1.100	0.876
	7	1	4.671	1.837	0.970	1.697	5.241	0.155	0.629	0.379	0.676	1.969	2.367	1.107	0.341	0.266
	8	1	7.587	1.476	4.646	1.200	5.580	0.509	0.690	0.151	1.179	1.512	1.202	1.190	0.884	0.286
REP4	1	1	2.305	3.354	3.214	2.077	5.844	0.066	0.239	0.265	0.284	1.437	1.190	1.978	0.387	0.709
	2	1	1.826	1.478	2.256	2.459	4.617	1.509	0.012	0.657	0.805	1.170	1.978	0.904	0.945	0.863
	3	1	3.723	1.493	2.021	3.507	5.589	0.315	0.022	0.298	0.085	1.912	0.904	2.521	1.310	0.860
	4	1	1.366	1.630	3.338	1.237	5.212	0.253	0.053	0.294	0.595	0.985	2.521	2.095	1.689	0.843
	5	1	1.559	2.874	1.663	1.872	6.105	0.327	0.051	0.396	0.232	0.439	2.095	2.471	4.202	0.701
	6	1	2.837	2.759	1.236	1.393	1.229	0.052	0.016	0.350	0.246	0.547	2.471	1.909	3.224	0.876
	7	1	2.169	1.767	1.945	3.025	4.092	0.155	0.198	0.221	0.173	1.018	1.909	2.190	0.561	0.452
	8	1	2.718	4.770	5.161	1.005	5.169	0.089	0.305	0.471	0.056	1.144	2.190	1.868	2.302	0.359
REP5	1	1	2.837	2.480	4.625	2.818	5.023	1.078	1.957	0.446	0.482	2.040	1.130	0.786	0.630	0.764
	2	1	2.169	3.026	5.542	2.280	4.945	0.291	0.207	0.483	0.016	1.416	1.763	1.398	2.737	0.043
	3	1	2.718	6.000	1.911	0.045	1.460	0.470	0.085	0.193	0.128	2.129	1.605	0.955	0.565	0.953
	4	1	2.643	2.630	1.944	1.125	0.884	0.039	0.014	0.199	0.357	1.111	1.587	0.675	1.043	0.717
	5	1	1.812	2.656	1.674	3.362	0.003	0.301	0.114	0.653	0.055	1.918	0.631	0.919	0.766	0.510
	6	1	0.962	1.332	1.709	1.270	1.961	0.016	0.118	0.638	0.461	0.059	0.588	0.905	1.737	0.562
	7	1	1.069	2.243	1.396	0.080	5.362	0.905	0.109	0.244	0.480	1.018	0.953	1.742	0.513	0.570
	8	1	1.569	3.894	1.759	1.159	0.402	1.071	0.107	0.217	0.464	0.902	1.078	1.107	0.324	0.608

(**E**)

Abundance of microDNA in cells (knockout and inhibited) treated with DSB inducing agents												
		DMSO	NCS	53BP1 + NCS	XRCC4 + NCS	NBS1+ NCS	POLQ+ NCS	MLH1+ NCS	MSH2+ NCS	PARP1i + NCS	B02+ NCS	D103+ NCS
REP1	1	1	2.872	5.820	5.098	0.852	0.624	0.568	0.604	1.474	1.104	2.746
	2	1	1.532	2.659	4.757	0.347	0.533	2.391	0.408	0.538	5.586	3.375
	3	1	2.367	4.180	6.612	0.658	0.535	0.282	2.402	1.521	2.601	1.665
	4	1	2.094	2.282	8.734	1.743	1.771	1.226	0.135	1.220	1.855	2.601
	5	1	2.234	4.152	5.603	1.263	0.592	0.558	0.815	1.354	1.066	1.445
	6	1	1.098	5.998	6.731	3.187	0.264	0.255	0.586	1.005	2.030	1.719
	7	1	2.396	6.719	5.851	1.413	0.468	0.464	0.506	1.907	1.137	3.492
	8	1	2.580	4.343	7.122	2.692	0.523	0.812	1.756	0.496	1.048	3.479
REP2	1	1	2.387	2.319	0.456	0.092	0.866	0.302	1.454	1.262	1.143	2.859
	2	1	1.712	5.129	1.808	1.807	2.725	1.216	4.945	1.739	2.601	0.071
	3	1	4.026	3.625	9.280	0.923	0.315	0.439	0.425	2.630	1.855	1.665
	4	1	2.606	4.152	11.148	0.110	1.482	0.334	1.775	0.950	1.066	3.375
	5	1	2.725	5.820	12.446	0.120	0.273	0.393	0.633	0.217	2.030	1.445
	6	1	2.947	4.180	7.039	0.562	0.541	1.382	1.370	1.351	1.137	1.719
	7	1	2.709	5.017	5.826	0.147	0.517	0.281	2.326	0.771	1.048	1.855
	8	1	2.523	5.751	12.117	0.138	0.502	0.850	1.474	0.813	1.115	3.479
REP3	1	1	1.993	6.719	8.786	1.789	0.045	2.665	1.459	0.171	1.105	2.494
	2	1	2.076	3.492	3.172	0.110	2.546	0.872	0.700	0.862	1.208	1.230
	3	1	2.650	8.072	3.646	1.913	3.756	0.691	4.179	0.496	0.846	2.228
	4	1	2.608	3.375	3.468	0.613	1.172	3.601	0.280	0.270	1.047	2.468
	5	1	3.001	7.055	6.513	1.245	2.051	0.330	0.532	0.199	1.403	3.155
	6	1	1.315	5.017	3.301	0.578	2.235	2.745	0.501	0.221	0.830	1.977
	7	1	1.051	4.343	1.732	1.891	2.776	3.003	0.527	0.205	1.461	3.218
	8	1	1.045	4.635	2.218	1.544	0.295	3.027	0.392	0.950	1.283	2.296

(**F**)

U2OS Knock-out, 293T small molecule inhibitors + PARP1i								
		U2OS	PARP1i	XRCC4+ PARP1i	MLH1+P ARPi	NBS1+ PARP1i	POLQ+ PARP1i	RAD51i+ PARP1i
REP1	1	1	0.359	0.263	0.754	1.490	0.297	0.562
	2	1	1.095	0.032	1.420	0.504	0.227	0.161
	3	1	0.452	0.046	0.666	0.311	0.122	0.062
	4	1	1.225	0.076	0.944	2.311	1.490	0.246
	5	1	0.286	0.203	0.212	0.523	1.286	0.582
	6	1	0.266	0.032	0.632	0.492	0.871	0.528
	7	1	0.288	0.249	0.944	1.832	0.299	0.571
	8	1	0.127	0.417	0.600	0.289	0.350	0.512
REP2	1	1	0.302	0.204	1.555	2.311	0.150	0.563
	2	1	0.442	0.432	1.116	3.169	1.584	0.408
	3	1	0.595	0.402	0.611	0.019	0.839	0.404
	4	1	0.513	0.121	0.649	0.105	0.034	0.021
	5	1	0.544	0.097	1.950	0.151	0.698	0.737
	6	1	0.527	0.111	0.772	0.081	0.200	0.547
	7	1	0.518	0.108	2.218	0.174	0.108	0.444
	8	1	0.608	0.103	0.598	0.237	0.200	0.456
REP3	1	1	0.363	0.323	1.677	1.413	1.205	0.412
	2	1	0.191	1.284	1.079	0.839	0.790	0.302
	3	1	0.240	2.630	1.478	0.147	1.174	0.570
	4	1	1.161	0.307	0.811	0.651	0.519	0.764
	5	1	0.729	0.263	0.600	0.202	1.533	0.043
	6	1	0.729	0.284	0.229	0.234	1.813	0.608
	7	1	0.270	0.267	0.693	0.171	1.494	0.337
	8	1	0.299	0.269	0.559	3.894	1.678	0.510

(**G**)

Abundance of MicroDNA through the Cell Cycle													
		DMSO	HU	HU REL 2.5	HU REL 5	HU REL 7.5	HU REL 9	NOC	NOC Rel 6	THY	THY REL 3	THY REL 6	THY REL 9
REP1	1	1	2.650	2.132	2.563	3.304	2.640	3.499	1.306	1.085	1.066	2.642	1.638
	2	1	2.232	2.974	1.155	2.738	0.460	4.216	1.002	2.178	0.733	1.441	2.146
	3	1	2.699	2.038	2.983	3.829	3.797	1.114	2.005	1.542	1.830	4.235	1.775
	4	1	2.044	2.693	0.268	0.241	3.884	3.115	0.691	1.214	1.284	3.730	1.994
	5	1	2.772	0.555	1.333	2.784	5.463	2.834	1.642	1.306	1.556	3.986	2.340
	6	1	0.939	3.260	2.831	5.945	4.604	3.615	0.249	1.120	1.550	3.578	1.553
	7	1	2.949	2.709	1.819	2.770	2.490	3.756	1.212	1.157	1.330	3.648	3.185
	8	1	0.798	1.945	1.763	2.717	1.769	2.479	1.814	6.082	1.079	3.979	2.180
REP2	1	1	2.584	2.132	0.857	0.679	1.812	2.814	2.749	1.310	1.304	1.173	4.053
	2	1	2.171	2.226	1.372	5.818	4.288	2.743	0.850	1.170	1.733	0.209	2.499
	3	1	2.477	1.409	2.258	4.421	4.880	2.985	1.025	2.314	2.373	1.433	0.920
	4	1	1.995	2.512	0.591	1.040	2.637	2.101	1.304	1.328	1.347	1.588	1.241
	5	1	2.874	2.421	3.772	0.965	2.490	4.405	2.033	1.301	1.359	1.495	0.412
	6	1	1.010	1.764	1.110	0.401	4.161	1.015	2.360	1.930	1.410	1.496	2.933
	7	1	0.693	3.118	0.990	3.240	2.415	2.192	0.645	2.784	1.368	1.580	4.664
	8	1	1.039	1.041	1.826	4.095	2.490	2.613	0.832	1.637	1.113	0.755	1.149
REP3	1	1	2.182	0.955	2.492	2.458	2.091	1.306	0.729	3.854	4.528	3.841	3.399
	2	1	0.989	0.448	3.884	2.016	0.586	4.144	1.379	0.460	1.230	1.051	1.798
	3	1	2.507	1.058	0.457	6.663	3.842	3.613	1.727	0.589	1.242	0.808	1.526
	4	1	1.711	2.330	1.548	1.911	2.092	4.306	0.745	1.405	0.912	0.683	0.865
	5	1	3.414	1.904	4.300	1.627	4.094	4.706	2.473	0.472	0.292	0.224	0.274
	6	1	1.339	0.973	1.250	1.968	1.961	2.749	1.530	2.039	1.865	2.923	2.352
	7	1	0.899	2.649	1.792	2.806	2.350	5.314	1.186	1.240	3.540	1.166	2.369
	8	1	0.724	0.899	1.885	0.749	2.236	1.814	0.444	1.385	0.895	0.615	0.839

Supplemental Table 3: Characteristics of the microDNA candidates in human cancer cell lines and DT40 cells. (A) Human cancer cell line microDNA candidates **(B)** DT40 cell line microDNA candidates. (microhomology between microDNA sequence and flanking genomic sequence indicated by arrows)

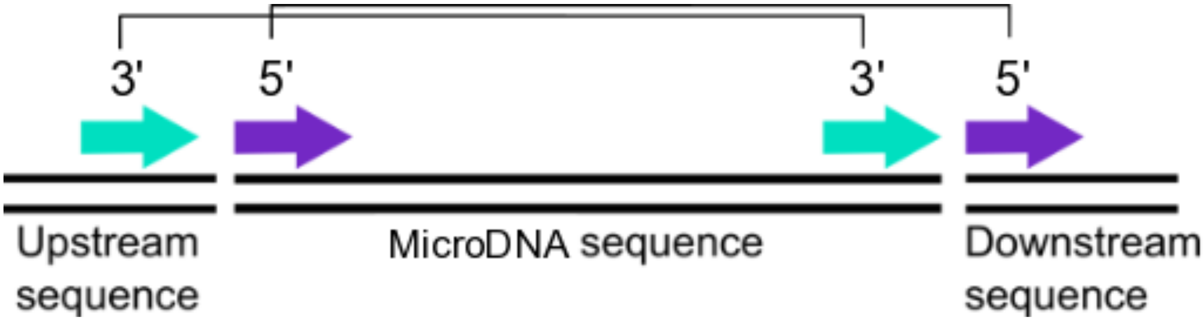

(**A**)

Human cancer cell lines MicroDNA candidates						
**#**	**Coordinates of microDNA by NGS**	**GC%**	**Genic sequence**	**Repeat region**	**Microhomology present in 20 bp of flanking region**	
					**5'**	**3'**
1	Chr1: 245530078-245530685	37.9	KIF26B	Partial LINE	TACA	AGAAAGG
2	Chr16: 83620011-83620259	59.3	CDH13	None	TGG	GGA
3	Chr12: 117100381-117100661	54.8	TESC-AS1	None	TT	TGC
4	Chr6: 43488800-43490545	41.4	TJAP1	None	CA	TGG
5	Chr14: 69346689-69347123	57.8	GALNT6	None	ACA	TCT
6	Chr11: 100650414-100650783	48.9	None	Partial SINE	TA	TG
7	Chr1: 245530303-245530648	40.7	KIF26B	None	AGC	AT
8	Chr19: 49091220-49091256	56.1	SNRNP70	None	CC	CT

(**B**)

DT40 cancer cell lines MicroDNA candidates						
**#**	**Coordinates of microDNA by NGS**	**GC%**	**Genic sequence**	**Repeat region**	**Microhomology present in 20 bp of flanking region**	
					5'	3'
1	Chr11: 2300736-2300969	42.7	CR406291	None	GA	GAT
2	Chr4: 1460467-1462710	53.3	DR416172	None	GC	CT

3	Chr3: 25576742-25577047	33	BX932473	None	AG	TA
4	Chr4: 82442326-82443624	36.6	None	None	ATT	TCA
5	Chr2: 69137047-69137904	35.4	None	None	TAT	TC
6	Chr8: 13132654-13133032	44.6	None	None	GGCAC	GA
7	Chr2: 20383321-20383744	43.9	X58519	None	ACC	AG
8	Chr1: 30701201-30701303	37.9	None	None	CAG	TA

Supplemental Table 4: Inverse PCR primers to detect microDNA formed from near a CRISPR-Cas9 induced double strand break on Chr22 and Chr12 and confirmation by sequencing that the amplicons were specific to the junctions.
Sequences of outward facing primers, size of inverse PCR amplicon and sequence of the amplicon with the two parts that are joined to form the circle are given. The two parts of the amplicon (on either side of the circularizing junction) are indicated by bold and regular font.

We believe that the microDNA does not contain the genomic region exactly up to the DSB because of (1) resection of the DNA after the DSB or (2) reconfiguration of the DNA during the repair.

EccDNA Induced by DSB: OF Primers and Amplicon Sequences					
#	UF Primer sequence	DF Primer Sequence	Coordinates of eccDNA by NGS	Size of Amplicon (bp)	Sequence of OF primer PCR amplicon
1	CCCAGTGCACCCCAACAAAGTGTTGAT	AGGGGAGGTGAGGGGTGAGTGAG	Chr22: 18,623,231-18,624,741	1225	**CTGGGCTGGGACCGTGGTCTGGATGCCAGGGAGTGGAAAGGAGCTGAAGGAAC**<br>**TCTCGCAGGCGGGCAGGCCTCCCAAGGGATTGGTTCAGCCAGAGAGGCAGGAC**<br>**ACGGACGGGCCAAGTCTCGGTGTGCATAGATTAGCGCTGGGGAAAGTCCCCGTG**<br>**GGGGCAGGCTGTGGGCAGGGAGGAGGGCTCACCAGCCAGCCAAGTGCCCGCAC**<br>**GAGGGAAGGAGTGGCCATCGTCCAGTGAGATGGTGAAGGGGACGTGGCCGCTC**<br>**TCATAGAGCAGGGGCGACACGAAGTGCACTTGCCCGGAGG**GTTTCACCACATTG<br>GCCAGGATGGTCTTGATCTCCTGACATCATCGTGATCCGCCCGCCTTGGCCTCCCA<br>AAGTGCTGGGATTACAGGCGTGAGCCACCGTAACCAGCCTGCCCTGCGTAATTTTT<br>GTTGTCGATGATCCAAAGTGCACCCACTCGCCATCTCTGGTAGCTGGGAGGAGCAG<br>GTTTTTGGGTGAAGGTGAAAGAGCCGGAGTTGGGGATGTGTGTGGCTGGGGGGTA<br>CAGGTGCGACCACTTTGCAGTCCACTCCTGTGAGTAGGGCATTCCTGAGGGAGAG<br>AAGGCCTGGTTAGCCCTGGGGCTCCTTCAAGGCCTGGCCCCAGGCCTTGATACCT<br>AGAGCAGCCTGACACATGTGTGGATGGACTGCCAGGCGGTCCCCTTAGGAGGGGC<br>TGTTCTAGCAGGGACCCTGGCTTGGGTGACCACCACCTCCTGCCACACTCCAGGA<br>TCCCTGTCCCTGCTGGAAGCCGGGCCCAGGGCCATGCCAGCCGTGAGGGGTTCC<br>TCATGAGCCTCCTCCCCGGGACCCAGGCCCCATGCAGCTGCCCCCTCCCACCTGC<br>CTGACCCCCCTGGCCTGCACTTCCCTCCATCCGAATCCTCAATCTCCGCCTGGAAT<br>TCCCTTCCCTCCTCAACCCCACCTGGAAACTCTCCTCCTCCTCCCACACAGCTGGG<br>ACTCCCTTTCTCCCACCTCTGGGGGCCCAAAGCTCTGGATGCCACCCTAGCCCTCA<br>GCTGGCCTCACCTGTCTCCTCATAGCCCCACAGCTTGATGGTGTCTGTGTGGGCAG<br>CGCCTCAACGTGCCAGGTCAGGCTGAGGTTGCCTGAGGTGTTGGCGGTGCCGTA<br>GTATTGCCAATGCATCTCGTTCATCAACTCACTCTTCTCCTTCATCAACACTTTGTTG<br>GGGTGCACTGGGCAGGG
2	CCAGTGCCTATCCACTGAGCTCCG	GTCCCAGCCCAGAGTGAGTGGG	Chr22: 18,624,003-18,624,948	680	**TGCTCTCCTGTGGCATGCAAACCTGTGGGCCTGGTGGTGGTAGGAGGTGCTGGG**<br>**CCACTGCAGGCTGAGCATCTCGACAGTCTGGGCCCCCAACCTCCCTGGGGAACC**<br>**CATCCAACCAGCTGTCCTGTGCCCTCCTGCCTGTCCTGGGTCTGAGGGGCTCCAT**<br>**TCAGCCTGCCAGTGGGTCTGAGCCCTCAGCATGCACAGGGATGGGGGCGTGGGG**<br>**CCCCAGTGCCCGGCCTCCTGGGCCTCCCAACCTGCAGATCAC**CCCTCGGCCATG<br>TGGACTCCTCCGGGCAAGTGCACTTCGTGTCGCCCCTGCTCTATGAGAGCGGCCA<br>CGTCCCCTTCACCATCTCACTGGACGATGGCCACTCCTTCCCTCGTGCGGGCACTT<br>GGCTGGCTGGTGAGCCCTCCTCCCTGCCCACAGCCTGCCCCCACGGGGACTTTC<br>CCCAGCGCTAATCTATGCACACCGAGACTTGGCCCGTCCGTGTCCTGCCTCTCTGG<br>CTGAACCAATCCCTTGGGAGGCCTGCCCGCCTGCGAGAGTTCCTTCAGCTCCTTTC<br>CACTCCCTGGCATCCAGACCACGGTCCCAGCCCAGAGTGAGTGGGAGCTGCAGG<br>GGTCCCTAGAGAGGTGGGCCAGTGCCTATCCACTGAGCTCCGCCACACCAGGGCA<br>GGGGAGAAGCCAGGTGG
3	CCCTCGGCCATGTGGACTCCTC	GCTCTCCTGTGGCATGCAAACCTGT	Chr22: 18624345-18625024	946	**GGGAGGTGAGGGGTGAGTGAGGGCTGGGCCCAGGGAAGCAGGGAGGAGGCCC**<br>**TGAGGCTTCAAGGGCTGTGGGAGCCTCCAGGGCCAATGCATGGCAGCAGCTGGG**<br>**TGATGGTCTCTCCGGCCCTCATGGCAGAAAAGGACGCTGAGGCCGGAGAGGCAG**<br>**GGGCCTGCCCTCCCTCCACTGCCCACGCCTCTAGCCTCCACCTGGCTTCTCCCCT**<br>**GCCCTGGTGTGGCGGAGCTCAGTGGATAGGCACTGGCCCACCTCTCTAGGGACC**<br>**CCTGCAGCTCCCACTCACTCTGGGCTGGGACCGTGGTCTGGATGCCAGGGAGTG**<br>**GAAAGGAGCTGAAGGAACTCTCGCAGGCGGGCAGGCCTCCCAAGGGATTGGTTC**<br>**AGCCAGAGAGGCAGGACACGGACGGGCCAAGTCTCGGTGTGCATAGATTAGCGC**<br>**TGGGGAAAGTCCCCGTGGGGGCAGGCTGTGGGCAGGGAGGAGGGCTCACCAGC**<br>**CAGCCAAGTGCCCGCACGAGGGAAGGAGTGGCCATCGTCCAGTGAGATGGTGAA**<br>**GGGGACGTGGCCGCTCTCATAGAGCAGGGGCGACACGAAGTGCACTTGCCCGG**<br>**AGGAGTCCACATGGCCGAGGGTCTGGATGCTCTCCTGTGGCATGCAAACCTGTG**<br>**GGCCTGGTGGTGGTAGGAGGTGCTGGGCCACTGCAGGCTGAGCATCTCGACAGT**<br>**CTGGGCCCCCAACCTCCCTGGGGAACCCATCCAACCAGCTGTCCTGTGCCCTCC**<br>**TGCCTGTCCTGGGTCTGAGGGGCTCCATTCAGCCTGCCAGTGGGTCTG**TGGTGTC<br>TGTGTGGGCAGCGCCTCAACGTGCCAGGTCAGGCTGAGGTTGCCTGAGGTGTTG<br>GCGGTGCCGTAGTATTGCCAATGCATCTCGTTCATCAACTCACTCTTCTCCTTCATC<br>AACACTTTGTTGGGGTGCACTGGGCAGGG
4	ATAGCGCCCACCAGCCTCTG	CCTGGAGAGGCACACAGCCA	Chr12:117100381-117100661	269	**ATAGCGCCCACCAGCCTCTGTGGCCATGTGCTGTGTCCACGCCAGGCCCTCTGGT**<br>**GGACATCTCATTAACTTATCCTTATTTTAAGGAGGAGGAATCACATTACACAGGCAA**<br>**CAGCATCGGCCCCAGTCACCCCAACTGGATGGAGA**AGACTAACGTTCAGAACAGA<br>CCTGATACCTGACGGTCATTGATGGTGGTGTCACTAAGCACTGCAGAGCCTTCAGG<br>AGAGCCTTGACCTTGATGTTCTGCCCTGGCTGTGTGCCTCTCCAGG

Supplemental Table 5: Characteristics of the loci used for CRISPR directed DSB experiment: The characteristics of the (**A**) genomic region where DSB was induced, (**B**) sgRNA target site and of the microDNAs detected, (**C**) microDNA amplified by outward facing primers, (**D**) DNaseI sensitivity.

(**A**)

Coordinates of regions probed by outward facing primers	GC%	Chromatin accessibility	Repeat region
Chr22: 18,623,992-18,634,095	60	DNaseI sensitive	Partial SINE, LINE
Chr12: 117,100,085-117,100,681	54	DNaseI sensitive	Partial SINE

(**B**)

Coordinates of gRNA homology	GC%	Chromatin accessibility	Repeat region
Chr22: 18,623,992-18,623,102	55	DNaseI sensitive	Partial SINE, LINE
Chr12: 117,100,085-117,100,105	55	DNaseI sensitive	Partial SINE

(**C**)

Coordinates of MicroDNA	GC%	Microhomology	Repeat region
Chr22: 18,623,221-18,624,741	63	None	Partial SINE
Chr22: 18,624,003-18,624,948	66	None	None
Chr22: 18,624,345-18,625,024	66	None	None
Chr12: 117,100,381-117,100,661	53	None	None

(**D**)

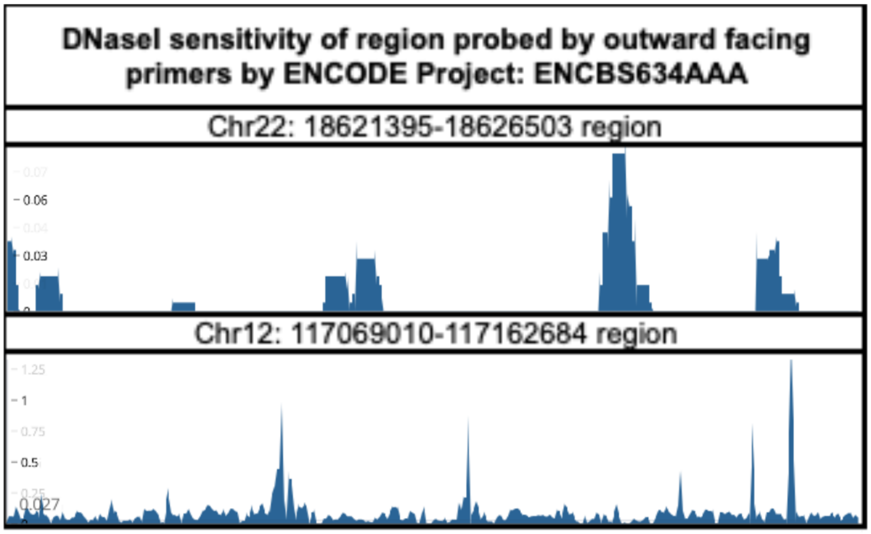
DNaseI sensitivity of region probed by outward facing primers by ENCODE Project: ENCBS634AAA
Chr22: 18621395-18626503 region
0.06
0.03
Chr12: 117069010-117162684 region
1
0.5
0.027

Supplemental Table 6:
The comparison of abundance of endogenous microDNA from hotspots (bottom 8 rows) with microDNA stimulated by DSB induction (rows labeled Chr22 gRNA).
The level of microDNA stimulated by the directly targeted DSB at Chr 22 was not as high as the most abundant endogenous microDNA from the hotspots 1 and 2 but were comparable to the microDNAs from the other hotspots.

The comparison of abundance of endogenous microDNA from hotspots (bottom 8 rows) with microDNA stimulated by DSB induction (rows labeled Chr22 gRNA).
The level of microDNA stimulated by the directly targeted DSB at Chr 22 (rows 4-6) was not as high as the most abundant endogenous microDNA from the hotspots 1 and 2 (rows 7,8) but were comparable to the microDNAs from the other hotspots (rows 9-14).

	MicroDNA category	Average abundance: eccDNA relative to mtDNA	Standard error: eccDNA relative to mtDNA
Control gRNA	DSB proximal eccDNA 1	2.72E-05	8.19E-06
	DSB proximal eccDNA 2	4.63E-08	2.01E-08
	DSB proximal eccDNA 3	1.55E-08	4.84E-09
Chr22 gRNA	DSB proximal eccDNA 1	4.78E-05	2.06E-05
	DSB proximal eccDNA 2	2.00E-07	7.44E-08
	DSB proximal eccDNA 3	5.97E-08	1.80E-08
MicroDNA arising from hot-spot	Candidate eccDNA 1	4.03E-02	1.18E-02
	Candidate eccDNA 2	1.04E-02	3.50E-03
	Candidate eccDNA 3	7.99E-07	3.86E-07
	Candidate eccDNA 4	4.69E-07	3.23E-07
	Candidate eccDNA 5	4.02E-07	1.69E-07
	Candidate eccDNA 6	9.05E-07	3.91E-07
	Candidate eccDNA 7	4.79E-07	2.70E-07
	Candidate eccDNA 8	2.30E-06	8.33E-07
